# Deep-Water Octocorals (Cnidaria, Anthozoa) from the Galápagos and Cocos Islands. Part 1: Suborder Calcaxonia

**DOI:** 10.3897/zookeys.729.21779

**Published:** 2018-01-16

**Authors:** Stephen D. Cairns

**Affiliations:** 1 Department of Invertebrate Zoology, National Museum of Natural History, Smithsonian Institution, P. O. Box 37012, MRC 163, Washington, D.C. 20013-7012, USA

**Keywords:** Octocorals, Galápagos, Cocos Islands, Calcaxonia, *Callogorgia*

## Abstract

Thirteen species of deep-water calcaxonian octocorals belonging to the families Primnoidae, Chrysogorgiidae, and Isididae collected from off the Galápagos and Cocos Islands are described and figured. Seven of these species are described as new; nine of the 13 are not known outside the Galápagos region. Of the four species occurring elsewhere, two also occur in the eastern Pacific, one off Hawaii, and one from off Antarctica. A key to the 22 Indo-Pacific species of *Callogorgia* is provided to help distinguish those species.

## Introduction

**Figure 1.**
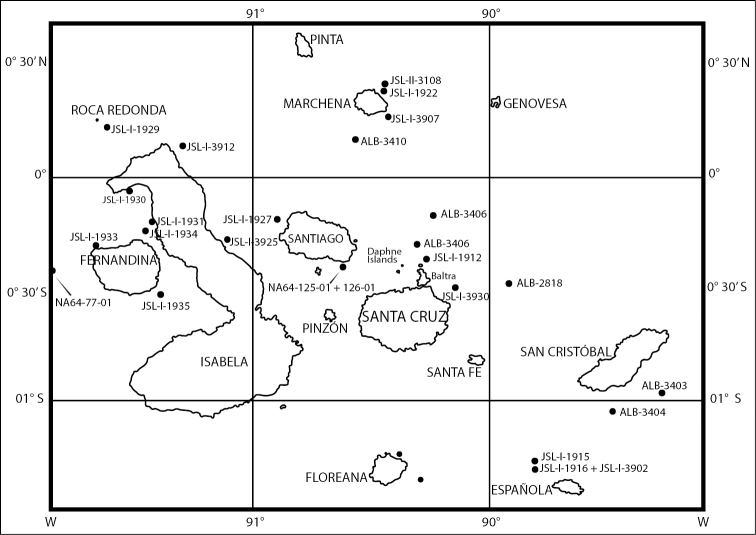
Map of the Galápagos Islands localities from where specimens are reported in this paper.

Early in my career (1986) I was privileged to participate in a deep-sea submersible expedition to the Galápagos and Cocos Islands, which was sponsored by SeaPharm, Inc. and HBOI (Pomponi et al. 1988). It made rich collections from 27 stations from off the Galápagos (*JSL-I*-1911 to 1937) and eight stations off the Cocos Islands (*JSL-I*-1938 to 1945) at depths to 823 m (Figure 1). Also on board this expedition were research scientists Shirley Pomponi and John Reed. Abundant deep-water invertebrates were collected at all stations, including many corals, but because the goal of the expedition was to seek biologically active compounds from these species, a 25-year sequestration was placed by the National Cancer Institute on all specimens collected. Nonetheless, as a participant in the expedition, I was given permission to publish on the Scleractinia (Cairns 1991a) and Stylasteridae (Cairns 1986, 1991b). Now, at the end of my career, I have come full circle to publish on the remaining deep-water corals collected by this expedition.

Although the 1986 *Johnson-Sea-Link* expedition was the first submersible expedition to sample the deep waters off the Galápagos, it was not the first to collect deep-water invertebrates from this region. As early as 1888 the *Albatross* had made 13 deep-water stations (*Alb* 2806 to 2819) off these islands, 14 more stations again in 1891 (*Alb* 3400–3413), and another 10 stations (*Alb* 4636–4646) in 1904, all these specimens being deposited at the NMNH. The *Johnson-Sea-Link* also made additional collections in the Galápagos in 1995 (*JSL-I*-3900 to 3985) and in 1998 (*JSL-II*-3084 to 3113). More recently the *E/V Nautilus* has made deep-water collections in July 2015. Deep-water octocorals from all of these cruises were examined to form a basis for this report.

Thirteen deep-water octocoral species are reported herein, i.e., those that belong to the suborder Calcaxonia: Primnoidae, Chrysogorgiidae, and Isididae. Approximately an equal number of deep-water octocorals belonging to the families Alcyoniidae, Acanthogorgiidae, Plexauridae and the order Pennatulacea have also been collected from these islands and will be reported in later parts. Of these thirteen species, seven are described as new, four have range extensions, and only two had been previously reported from the Galápagos by Studer (1894).

## Material and methods

As explained in the Introduction, this study is based on all the deep-water calcaxonian octocoral specimens collected by the *Albatross* and *John-Sea-Link I* and *II*, which are deposited at the NMNH. It also includes material collected by the *Nautilus*, which is deposited at the CDRS.

The methodology concerning sclerite preparation for SEM can be found in Cairns (2016). SEM stub numbers are in a series established by the author, and all are deposited at the NMNH. Morphological terminology follows the glossary of Bayer et al. (1983), as updated by Cairns (2012, 2016).

The following abbreviations are used in the text:

**Museums / institutions**

**BM** British Museum (now The Natural History Museum, London)

**CDRS** Charles Darwin Research Station, Galápagos

**HBOI** Harbor Branch Oceanographic Institute, Ft. Pierce, Florida

**NMNH** National Museum of Natural History, Smithsonian, Washington DC

**USNM** United States National Museum (now the NMNH)

**Vessels**

***Alb*** USFWS *Albatross*

***GS*** R/V *Gilliss*

***JSL-I*** *Johnson-Sea-Link I* submersible (HBOI)

***JSL-II*** *Johnson-Sea-Link II* submersible (HBOI)

***NA*** *E/V Nautilus*

**Other terms**

**L:W** ratio of length to width of a polyp or sclerite

**SEM** scanning electron microscope

## Systematic account

### Subclass Octocorallia

#### Order Alcyonacea

##### Suborder Calcaxonia

###### Family Primnoidae Milne Edwards, 1857

####### Genus Callozostron

Taxon classificationAnimaliaAlcyonaceaPrimnoidae

Wright, 1885

Callozostron Wright 1885: 690–691; Wright and Studer 1889: 48; Bayer 1996: 151–152; Cairns and Bayer 2009: 32–33; Cairns 2016: 26–27 (key to species).

######## Type species.

*Callozostron mirabile* Wright and Studer, 1889, by monotypy.

######## Diagnosis.

Colonies unbranched, sparsely dichotomously branched, or pinnately branched. Polyps arranged in whorls, the bases of adjacent polyps sometimes fused. Polyps covered by eight longitudinal rows of body wall scales, which completely cover the polyp. At least four and up to 24 marginal and submarginal scales bear elongate slender apical spines; marginal scales do not fold over operculars. Coenenchymal scales similar to those of body wall, arranged in one layer. All scales thin, with a smooth pouter face and tuberculate inner face.

######## Distribution.

Antarctic, Subantarctic, Antipodes, New Zealand, Clarion-Clipperton Fracture Zone, Galápagos, 744–4235 m deep.

######## Remarks.

A discussion and key to the six species in this genus are given by Cairns (2016).

####### Callozostron carlottae

Taxon classificationAnimaliaAlcyonaceaPrimnoidae

Kükenthal, 1909

Figures 3h , 4

Callozostron carlottae Kükenthal 1909: 49–50; 1912: 334–336, pl. 22, figs 14–17, text-figs 43–47. Not: Bayer 1996: 159–161, figs 7–10 (but includes a complete synonymy); Cairns 2016: 27.

######## Material examined.

*Nautilus* NA064-77-01-A, 1 branch, 1.82° C, CDRS, and SEM stubs 2376–2378, NMNH.

######## Types.

Four specimens (?syntypes) are included in the original description. Their deposition is unknown.

######## Type locality.

Antarctica (between 60–90°E), 3397 m depth (*Gauss* German South Polar Expedition of 1901–1903).

######## Distribution.

Galápagos: east of Fernandina, 3381 m depth. Elsewhere: Antarctica, 3397 m depth.

######## Description.

Although only one incomplete colony is available (Figure 3h), it appears to be unbranched and 9 cm in length. Its polyps are arranged in whorls of six or seven (Figure 4a), about five whorls occurring per cm branch length; the whorl diameter is about 4 mm, including the marginal spines. The polyps are short and cylindrical (Figures 4b-d), only about 1.2–1.4 mm in height, not counting the marginal spines, which can be up to twice as long as the polyp. The actual body wall is relatively short (about 0.5 mm), the opercular scales making up the remaining length of the polyp.

The body wall scales (Figures 4f-i) are arranged in eight irregular longitudinal rows, the two distal marginal and submarginal body wall scales being quite different from the lower four or five tiers of scales, the latter (Figure 4f) being roughly elliptical to rectangular in shape, quite thin, and often having a slightly lobate distal margin. The body wall scales are slightly curved to fit the circumference of the polyp and are highly imbricate. Their outer face is perfectly smooth, their inner face covered with sparse small tubercles. There are eight marginal and usually eight submarginal scales. The marginal scales (Figures 4g, i) have a rectangular to trapezoidal base that is up to 0.6 mm in width, capped by a slender (0.1 mm in diameter basally, but attenuating to a point distally) elongate (up to 2.2 mm in length) spine, the spinose part thus contributing 75–80% of the length of the sclerite. The spine is smooth but itself covered with very small (about 15 µm in length) spines. The submarginal scales (Figure 4h) may be as large as the marginal scales, but some are only about half as long (0.75–0.90 mm in length, see below). The eight operculars (Figures 4b, e) are arranged in two alternating quartets of four, an inner quartet and an outer, the lateral edges of the outer operculars overlapping those of the inner. The opercular scales are isosceles triangular in shape with a pointed (not spinose) tip, and having a longitudinally concave, perfectly smooth outer surface and a sparsely tuberculate inner surface. They are slightly curved about 45°in order to fold over the polyp to form the operculum. The operculars are 0.7–1.0 mm in height, with a L:W of 1.9–2.6. The marginal and submarginal scales that are aligned with the four inner opercular scales are usually both large in size, whereas the submarginals associated with the outer opercular scales are often much smaller (see above) and may even be absent. The coenenchymal scales (Figure 4j) are irregular in shape but usually elongate, up to 0.65 in greater length. Like most of the other scales, they have a smooth outer surface and a sparsely tubercular inner surface, and are quite thin, and easily broken.

######## Comparisons.

Despite the long distance between the Antarctic type locality and the Galápagos, this specimen is identified as *C. carlottae*, but it does differ in several points from the original description. The Galápagos specimen has larger polyps, and thus larger opercular and marginal spines, those of the Antarctic syntypes being only 0.6 mm and 0.8 mm in length, respectively. And the Antarctic syntypes have eight or nine polyps per whorl, whereas the Galápagos specimen has only six or seven. Otherwise, the two specimens are remarkably similar, the Galápagos specimen even showing the dimorphic-sized submarginal spines (Kükenthal 1912: fig. 43). The specimens reported by Bayer as *C. carlottae* are not considered conspecific, based on a difference in the size and number of rows of submarginal spines (see Cairns 2016), as well as having differently shaped marginal spines and thicker granular body wall scales.

######## Remarks.

This is the first report of this species subsequent to its original description, and was collected at virtually the same depth.

####### Genus Callogorgia

Taxon classificationAnimaliaAlcyonaceaPrimnoidae

Gray, 1858

Callogorgia Gray 1858: 286; Bayer 1982: 119–123; 1998: 162–163; Cairns and Bayer 2002: 841–845; 2009: 40; Cairns 2010: 425; 2016: 58.
Caligorgia Wright and Studer 1889: 75–77; Versluys 1906: 55–58; Kükenthal 1919: 362–366.

######## Type species.

*Gorgonia verticillata* Pallas, 1766, by monotypy.

######## Diagnosis.

Colonies uniplanar, pinnately or dichotomously branched. Polyps cylindrical to clavate, arranged in whorls of up to 12, all polyps facing upward. Polyps covered with eight longitudinal rows of body wall scales, the number of scales per row decreasing from ab- to adaxial polyp side. Body wall scales granular, smooth, pitted, or covered with tall ridges (cristate). Inner side of opercular scales convex, covered with a multiply serrate keel.

######## Distribution.

Indo-Pacific, Atlantic, 37–2472 m deep.

######## Remarks.

The identification of the species of *Callogorgia* has been greatly facilitated by the availability of previously published dichotomous keys. The structure and characters used for the first published key were chosen by Kükenthal (1919) and later refined by Kükenthal (1924) and Bayer (1982). Since Bayer’s key, which was limited to the Indo-Pacific species, at least one species (*C. cristata* (Aurivillius, 1931)) has been synonymized, two (*C. ventilabrum* Studer, 1878, *C. laevis* Thomson and Mackinnon, 1911) transferred to different genera, four described as new (*C. tessellata* Cairns, 2016, *C. dichotoma* Cairns, 2016, *C. galapagensis* sp. n., *C. arawak* Bayer et al., 2014), two previously overlooked (*C. gilberti* (Nutting, 1908), *C. modesta* (Studer, 1878)), and the characteristics of *C. kinoshitai* (Kükenthal, 1913) re-evaluated. Thus, a revised key is provided below, which relies heavily on the characters used in previously published keys. *C. dubia* (Thomson and Henderson, 1906), based on a poor description and no figures, is of doubtful attribution and is not considered in the key. Previously *C. elegans* (Kükenthal 1919, Bayer 1982) had been keyed as having 12–13 abaxial scales, but examination of the type in the BM by Bayer (pers. comm.) indicates that it has only six to eight abaxial body wall scales and a body wall sclerite formula of: 6–8:2:0:1. Keys to the five western Atlantic species were published by Cairns and Bayer (2002) and Bayer et al. (2014). Altogether, there are currently 28 valid species in the genus (Cairns and Bayer 2009; Taylor and Rogers 2015).

###### Key to the 22 Indo-Pacific species of *Callogorgia* (species included herein in bold face; species having ridged (cristate) abaxial body wall scales indicated with an asterisk)

**Table:** 

1	Branching typically pinnate	**2**
–	Branching dichotomous or quasi-dichotomous	**13**
2	Branches strictly opposite	***C. formosa* (Kükenthal, 1907)**
–	Branches alternate	**3**
3	Scales in outer lateral body wall rows well developed, i.e., having the same or only slightly fewer than those in abaxial row	**4**
–	Scales in outer lateral body wall rows sharply reduced in number, i.e., usually less than half the number as that in abaxial row	**6**
4	Coenenchymal scales elongate and not thick; outer surface of abaxial body wall scales on distal half of polyp highly ridged	*C. galapagensis sp. n.
–	Coenenchymal scales irregular in shape and quite thick (tessellate); outer surface of body wall scales granular or pitted (not ridged)	**5**
5	Seven to nine scales in abaxial body wall scale rows; body walls scales pitted; polyps 1.4–1.8 mm in length	***C. sertosa* (Wright and Studer, 1889)**
–	Nine to eleven scales in abaxial body wall scale rows; body wall scales granular; polyps 1.0–1.2 mm in length	***C. tessellata* Cairns, 2016**
6	Operculum low and inconspicuous	***C. pennacea* (Versluys, 1906)**
–	Operculum tall and prominent	**7**
7	Abaxial opercular scales with two to four apical points	*C. weltneri (Versluys, 1906)
–	Abaxial opercular scales with a single point	**8**
8	Eight or more scales in each abaxial body wall row	**9**
–	Six to eight scales in each abaxial body wall row	**11**
9	Apex of opercular scales prolonged into a rod-like (cylindrical) point	*C. ramosa (Kükenthal and Gorzawsky, 1908)
–	Apex of opercular scales not prolonged in a cylindrical point	**10**
10	Eight to ten scales in abaxial body wall scale rows	*C. flabellum (Ehrenberg, 1834)
–	Eleven to thirteen scales in abaxial body wall scale rows	*C. gilberti (Nutting, 1908)
11	Polyps about 2 mm tall	*C. robusta (Versluys, 1906)
–	Polyps 1.0–1.8 mm tall	**12**
12	Coenenchymal scales elongate; inner lateral and adaxial body wall scales present; eastern Pacific	***C. kinoshitai* (Kükenthal, 1913)**
–	Coenenchymal scales polygonal; inner lateral and adaxial body wall scales absent; South Pacific	***C. joubini* (Versluys, 1906)**
13	Scales in outer lateral body wall rows well developed, i.e., having the same or only slightly fewer than those in abaxial row	**14**
–	Scales in outer lateral body wall rows sharply reduced in number, i.e., usually less than half the number as that in abaxial row	**15**
14	Five or six scales in each abaxial body wall row	***C. dichotoma* Cairns, 2016**
–	Ten scales in each abaxial body wall row	***C. versluysi* (Thomson, 1905)**
–	Twelve or 13 scales in each abaxial body wall row	***C. imperialis* Cairns, in press**
15	Three scales in each outer lateral body wall row	**16**
–	One or two scales in each outer lateral body wall row	**17**
16	Four polyps per whorl	***C. elegans* (Gray, 1870)**
–	Two or three polyps per whorl	***C. indica* (Thomson and Henderson, 1906)**
17	Five scales in each abaxial body wall row	**18**
–	Seven scales in each abaxial body wall row	**19**
18	Two or three polyps per whorl; eight to nine whorls per cm branch length; body wall scales with low marginal ridges	***C. minuta* (Versluys, 1906)**
–	Three or four polyps per whorl; 9–11 whorls per cm branch length; body wall scales with distal margins strongly reflexed, exposing high crest-like ridges on inner face	***C. chariessa* Bayer, 1982**
19	Two or three polyps per whorl; seven to eight whorls per cm branch length	***C. similis* (Versluys, 1906)**
–	Four to six polyps per whorl; nine to ten whorls per cm branch length	**20**
20	Abaxial body wall scales with strong marginal ridges	*C. affinis (Versluys, 1906)
–	Abaxial body wall scales with only weak marginal ridges	***C. modesta* (Studer, 1878)**

####### Callogorgia galapagensis sp. n.

Taxon classificationAnimaliaAlcyonaceaPrimnoidae

http://zoobank.org/F21CD90F-41C8-4A0A-9B2B-6CF8E4E9E6A4

Figures 2a , 5

######## Material examined.

**Types**. Holotype: *JSL-I*-1933, large colony and SEM stubs 2295–2297, 2308–2311, USNM 1161744. Paratypes: *JSL-I*-1915, partial colony, USNM 1161750; *JSL-I*-1930, partial colony, USNM 1161746; *JSL-I*-1934, 1 branch, USNM 1161745; *JSL-I*-1942, 1 branch, USNM 1161748.

######## Type locality.

*JSL-I*-1933: 0°17.072'S, 91°04.208'W (off northwest tip of Fernandina, Galápagos), 663–788 m deep.

######## Distribution.

Galápagos: Tagus Cove between Isabela and Fernandina, north of Española, 308–633 m deep; Cocos Island, 628–656 m deep.

######## Description.

**Figure 2.**
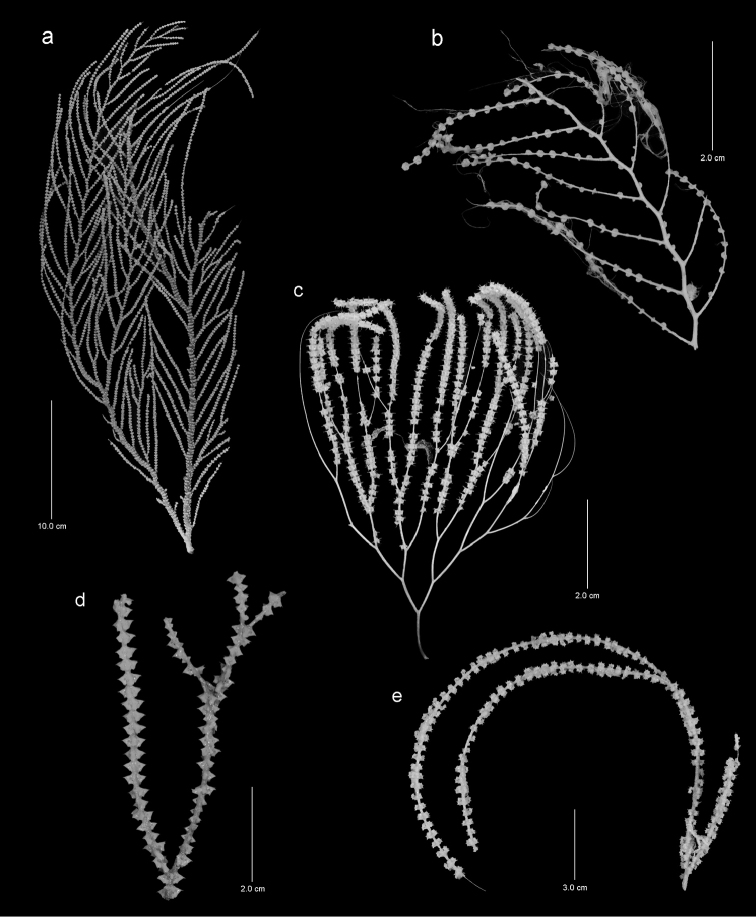
Colonies of various species. **a** *Callogorgia galapagensis*, holotype, USNM 1161744 **b** *Callogorgia kinoshitai*, *Alb*-3406, USNM 50960 **c** *Calyptrophora agassizii*, *JSL-II*-3108, USNM 1093041 **d** *Calyptrophora reedi*, holotype, USNM 1409027 **e** *Narella ambigua*, *JSL-I*-1927, USNM 1297223.

Colonies are uniplanar and taller than broad, the holotype (Figure 2a) measuring 49 cm in height and 18 cm in width, with a broken basal branch diameter of 5.9 mm. Another colony fragment (*JSL-I*-1915) has a broken basal branch diameter of 8.9 mm, suggesting a colony height of close to 1 m. Branching is alternate pinnate (sympodial and geniculate), the terminal branchlets up to 13 cm in length. The polyps are arranged in whorls of five or six (Figure 5a); four to five whorls occur per cm branch length; the whorl diameter ranges from 2.5–3.1 mm. The polyps are 1.5–2.0 mm in length, slightly curved, and clavate (Figure 5b–d). The color of the colony and polyps is white.

There are eight longitudinal rows of body wall scales, decreasing in number from ab- to adaxial polyp side, the body wall sclerite formula being: 10–12:10–12:4–5:2. The distal five or six pairs of abaxial scales (Figure 5f) are narrow (0.31–0.35 mm wide), each bearing four to seven prominent (up to 0.08 mm in height) longitudinal ridges that terminate as projections on the distal edge of the scale. More proximal abaxial body wall scales are broader (up to 0.48 mm) and flat, lacking radial ridges. The outer lateral body wall scales (Figure 5h) are sculpted similarly, the basal pairs being even wider than those on the abaxial face. The ridges of these distal scales are so tall and closely spaced that it is difficult to determine the lateral margins of the scales. The inner lateral body wall scales (Figure 5g) are even broader (up to 0.56 mm in width) and have a finely serrate distal edge. The two pairs of adaxial scales (Figure 5i) are small, only 0.22–0.25 mm in width, below which the polyp is naked (Figure 5c). At the junction of the lowest body wall scales and the branch coenenchymal sclerites is a pair of crescentric infrabasal scales (Figure 5j) that forms a transition, each about 0.6 mm wide and 0.25 mm in height. The distalmost body wall scales in each row fold over the operculum as a short circumoperculum (Figure 5c). The opercular scales (Figure 5e) range in length from 0.50–0.65 mm, decreasing in length from ab- to adaxial polyp side, forming a prominent operculum; their L:W ranges from 1.7–2.25. Their outer surface is covered with tall serrate ridges and their edges are finely serrate. Their inner face is tuberculate, the distal third bearing a multiply serrated keel. The coenenchymal sclerites (Figure 5k) are elongate (L:W = 5–6), thick sclerites, arranged parallel to the branch axis, measuring up to 0.8 mm in length and 0.13–0.14 m in width. Their outer surface is coarsely granular.

**Figure 3.**
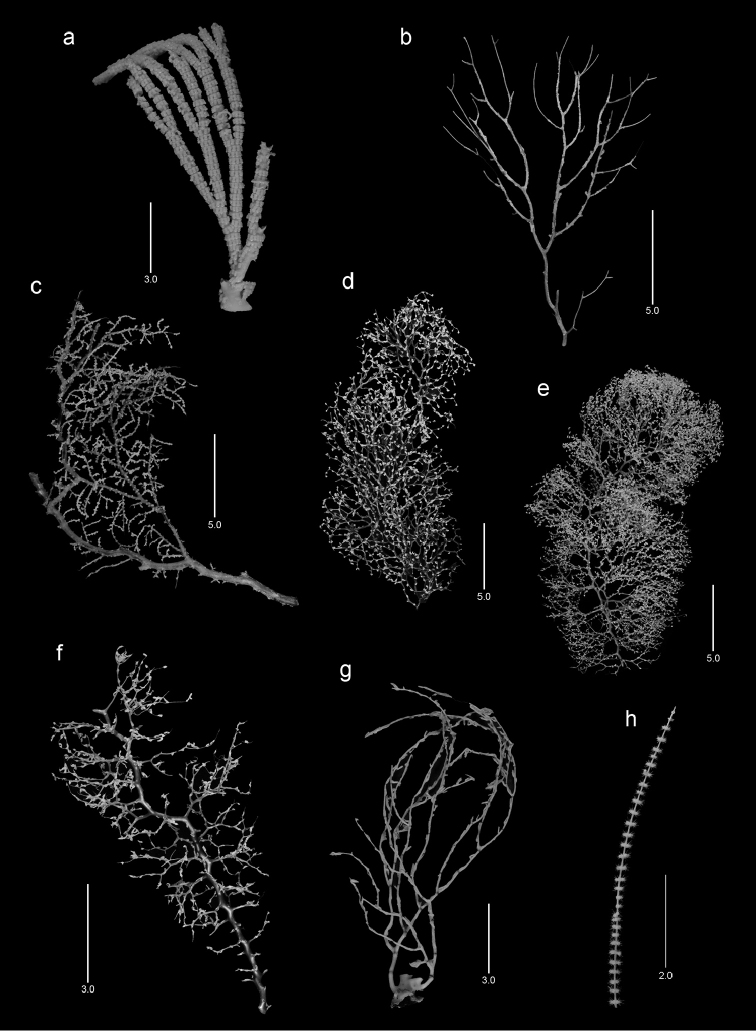
Colonies of various species. **a** *Narella enigma*, holotype, USNM 1409703 **b** *Plumarella abietina*, *Alb*-2818, USNM 49605 **c** *Parastenella pomponiae*, holotype, USNM 1410289 **d** *Chrysogorgia scintillans*, *JSL-II*-1927, USNM 89377 **e** *Chrysogorgia midas*, holotype, USNM 1160575 **f** *Chrysogorgia laevorsa*, holotype, USNM 1409029 **g** *Isidella tenuis*, holotype, USNM 89382 **h** *Callozostron carlottae*, *NA*64-77-01-A.

**Figure 4.**
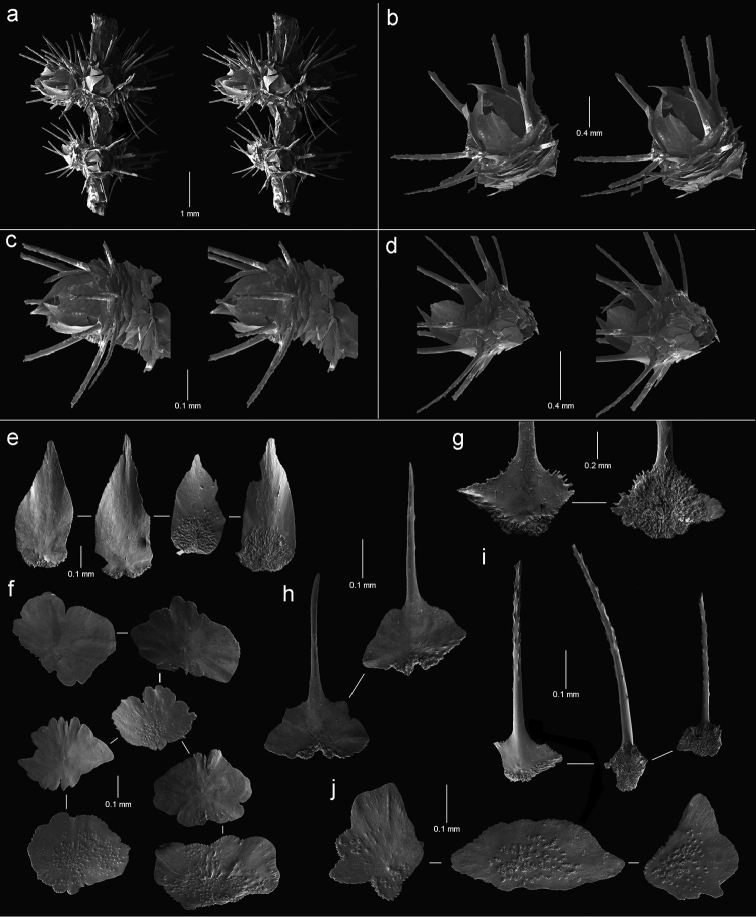
Polyps and sclerites of *Callozostron carlottae* from *NA*64-77-01-A. **a** lateral stereo view of two polyp whorls **b–d** lateral stereo views of three polyps showing spinose marginal and submarginal scales and non-spinose proximal body wall scales **e** opercular scales **f** lower, non-spinose body wall scales **g** wide base of marginal scales **h** spinose submarginal scales **i** marginal scales **j** coenenchymal scales.

######## Comparisons.

*Callogorgia galapagensis* belongs to a group of eight species that have highly cristate abaxial body wall scales, the other seven species listed in Cairns (2016), the Pacific component indicated in the key above by asterisks. The prominent ridges on these body wall scales often make it difficult to see the boundaries between adjacent rows of body wall scales. *Callogorgia galapagensis* can be distinguished from the other seven species by its sclerite formula, being the only species to have 10–12 abaxial and outer lateral body wall scales. This character is not used in the key above, and thus *C. galapagensis* keys closest to *C. sertosa* and *C. tessellata*, but can be distinguished by its unique sclerite formula.

**Figure 5.**
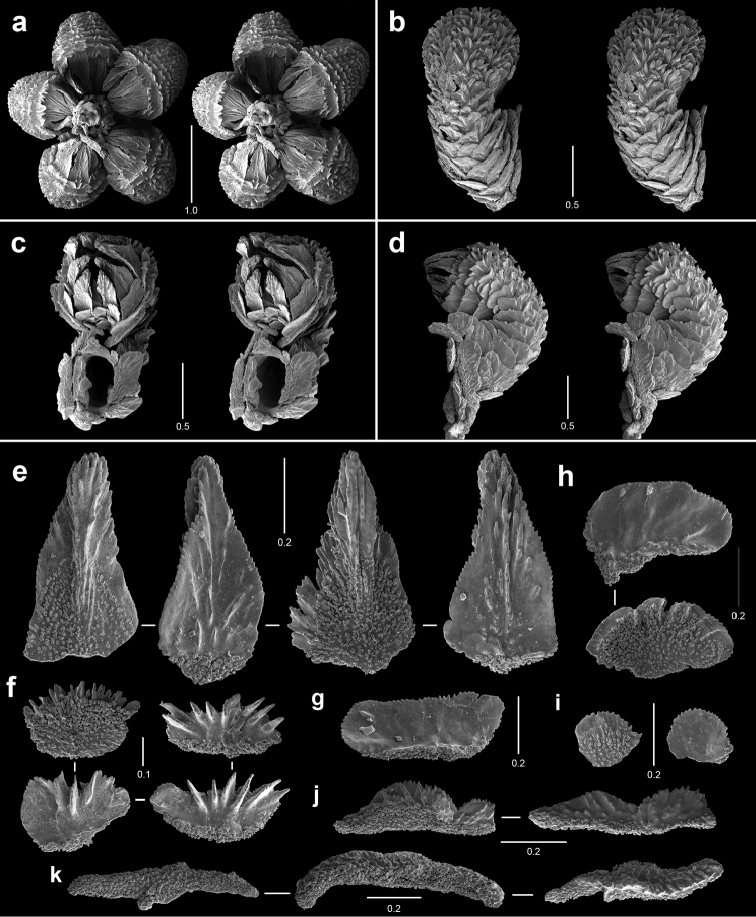
Polyps and sclerites of *Callogorgia galapagensis* from the holotype, *JSL-I*-1933, USNM 1161744. **a** apical stereo view of polyp whorl **b–d** abaxial, adaxial, and lateral stereo views of a polyp, respectively **e** opercular scales **f** abaxial body wall scales **g** inner lateral body wall scale **h** outer lateral body wall scales **i** adaxial body wall scales **j** infrabasal scales **k** coenenchymal scales.

######## Etymology.

Named for the type locality of the species.

####### Callogorgia kinoshitai

Taxon classificationAnimaliaAlcyonaceaPrimnoidae

(Kükenthal, 1913), nom. correct.

Figures 2b , 6

Calligorgia sertosa Nutting 1909: 715.
Calligorgia kinoshitae Kükenthal 1913: 264–266, text-figs E–F, pl. 8, fig. 10; Kükenthal 1919: 370; 1924: 270.
Callogorgia kinoshitae Bayer 1982: 122 (key); Cairns 2007b: 512 (listed); Cairns and Bayer 2009: 29 (listed).

######## Material examined.

*Alb*-3406, 1 colony and SEM stubs 2300–2302, USNM 50960; *Alb*-3410, 1 colony, USNM 50959; *Alb*-4530, 1 colony, USNM 49611; *Alb*-4537, 3 colonies, USNM 30030, 43126, and 58986; *Desteiquer*, 36°37'12"N, 122°13'18"W, 1476–1609 m, 1 colony, USNM 75231; *Alb*-4357, USNM 30084 (syntype).

######## Types.

Kükenthal (1913) established this species on the specimens described by Nutting (1909) as *C. sertosa*. Although Kükenthal examined some of those specimens and added information about them, he did not report any additional specimens or designate a type. The specimens reported by Nutting (1909) are thus considered as syntypes, and include those from *Alb*-4356, *Alb*-4357 (USNM 30084, SEM stubs 2298–2299), *Alb*-4358, *Alb*-4386, and *Alb*-4391, all presumably deposited at the NMNH, although only *Alb*-4357 could be located in 2017.

######## Type locality.

As defined by the syntype series, the type-locality extends from northern Baja California (latitude 30°30'30"N) to just north of San Diego (latitude 33°02'15"N), and includes the bathymetric range of 219–2469 m.

######## Distribution.

Galápagos: between Santa Cruz and Marchena, 605–1008 m deep. Elsewhere: northern Baja California to Monterey Bay (new records), California, 219–2469 m deep.

######## Description.

**Figure 6.**
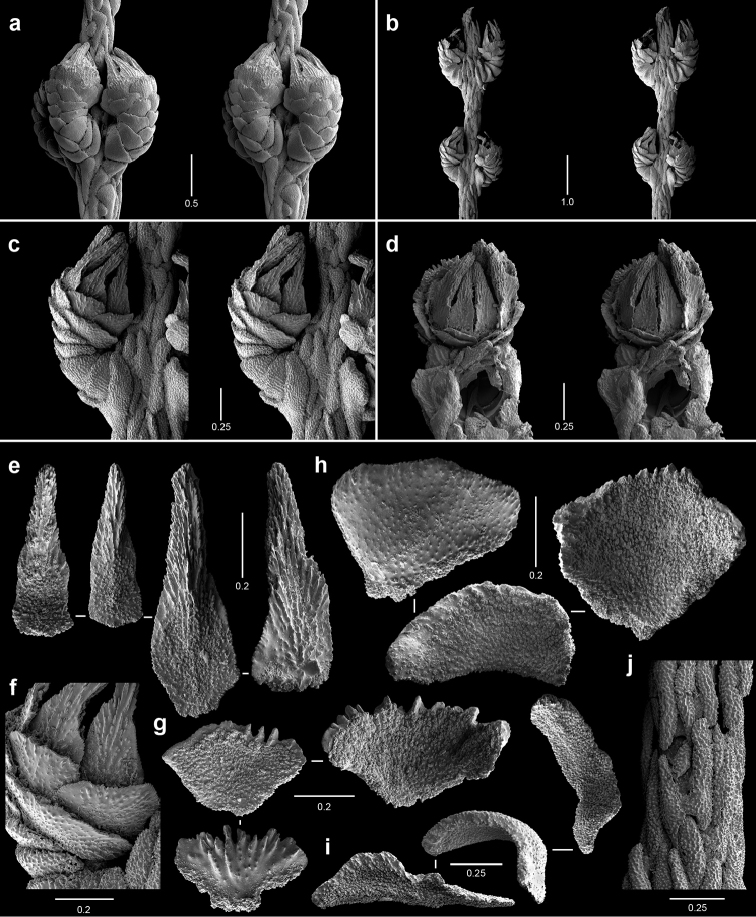
Polyps and sclerites of *Callogorgia kinoshitai* (**a** *Alb*-4357, USNM 30084 **b–j** *Alb*-3406, USNM 50960) **a–b** lateral stereo views of polyp whorls **c** lateral stereo view of a polyp **d** adaxial stereo view of a polyp **e** opercular scales **f** view of outer lateral scales, in situ **g** marginal scales **h** abaxial body wall scales **i** infrabasal scales **j** coenenchymal scales, in situ.

Colonies are uniplanar and taller than wide, the largest Galápagos specimen (*Alb*-3406) a broken colony only 18 cm in height, but the largest syntype measuring up to 26 cm in height. Branching is alternate pinnate (sympodial and geniculate, Figure 2b), the terminal distal branchlets only about 4 cm in length. The polyps are arranged in whorls of two or three (Figures 6a, b), although Kükenthal (1919) reported a range of two to six, with four being the most common number. The whorl diameter is about 1.5 mm. The polyps are 1.3–1.5 mm in length, and slightly clavate (Figures 6c, d). The color of the colony and polyps is white.

There are eight longitudinal rows of body wall scales, decreasing in number from ab- to adaxial polyp side, the body wall sclerite formula being: 6–8:1–2:1–2:1–2. The marginal and submarginal abaxial body wall scales (Figure 6g) have a ctenate distal edge and increase in width and thickness from distal to proximal position. The basalmost abaxials are short but quite wide (up to 0.95 mm), curving around most of the basal part of the polyp and occupying the space normally reserved for the proximal outer and inner lateral scales. These scales serve as infrabasals (Figure 6i), forming a transition from the plate-like body wall scales (Figure 6h) to the thick elongate coenenchymal scales (Figure 6j). The outer (Figure 6f) and inner lateral body wall scales are quite wide (0.5–0.6 mm) and have a finely serrate distal edge. The adaxial scales (Figure 6d) are narrow, only about 0.3 mm in width. The outer surface of the body wall scales is relatively smooth, and covered with small, low granules. All body wall scales are curved to fit the circumference of the polyp body. The opercular scales (Figure 6d, e) range in length from 0.50–0.75 mm, decreasing in length from ab- to adaxial polyp side, forming a prominent operculum; their L:W ranges from 2.8–3.0. The relatively high L:W ratio is caused by an attenuate tip that is essentially round in cross section and covered on all surfaces by small spines arranged in longitudinal rows. The coenenchymal sclerites (Figure 6j) are elongate (L:W = 5–6) and thick, up to 0.55 mm in length and about 0.1 mm in width. They are longitudinally arranged in one layer on the branches. Their outer surface covered by low granules.

######## Comparisons.

Although this species was originally identified as *C. sertosa* by Nutting (1909), that species differs in having more pairs of outer body wall scales, a pitted body wall outer surface texture, and a tessellate coenenchymal scale arrangement (Cairns 2016). As seen from the key above, *C. kinoshitai* is most similar to *C. joubini* (Versluys, 1906) (Indonesia, 520 m deep) but differs from that species in having inner and outer lateral scales and elongate (not polygonal) coenenchymal scales.

######## Remarks.

Kükenthal (1919) described a sclerite formula of 7–8:7–8:4:2 for this species, but the syntype from *Alb*-4357 clearly has only two outer lateral scales and thus his sclerite formula is doubted.

######## Nomenclature.

This species was clearly named after Kumao Kinoshita, and thus the name is herein changed to reflect a masculine ending.

####### Genus Calyptrophora

Taxon classificationAnimaliaAlcyonaceaPrimnoidae

Gray, 1866

Calyptrophora Gray 1866: 25; Bayer 2001:267–368; Cairns 2007b: 512 (listed); Cairns and Bayer 2009, 44–45; Cairns 2009: 420–426 (key to species); Cairns 2012: 42–43 (key to New Zealand species).

######## Type species.

*Calyptrophora japonica* Gray, 1866, by monotypy.

######## Diagnosis.

Colonies uniplanar to slightly bushy (lyrate, dichotomous, polychotomous, biplanar) or unbranched. Polyps arranged in whorls, the polyps facing either upward or downward. Polyps consist of two annular sclerite rings, each composed of two inseparably fused scales; a pair of crescent-shaped infrabasals also present. Articular ridge present between basal and buccal body wall scale. Distal margins of body wall scales often spinose, toothed, or lobate. Operculum composed of eight scales; keels usually present on inner face of opercular scales. Coenenchymal scales elongate and flat, sometimes quite thick (as plates). Small curved tentacular platelets often present.

######## Distribution.

Tropical and temperate latitudes of Atlantic, Pacific, and Indian Oceans, 227–3531 m deep.

######## Remarks.

Including the new species described herein, the genus contains 23 species, making it the fifth most species-rich genus within the primnoids. Bayer (2001) divided the genus into two species complexes, one having their polyps directed upward, the other having their polyps directed downward. Although helpful in grouping and identifying species, these two species groups do not seem to have phylogenetic validity (Cairns 2009, Cairns and Wirshing in review).

####### Calyptrophora agassizii

Taxon classificationAnimaliaAlcyonaceaPrimnoidae

Studer, 1894

Figures 2c , 7

Calyptrophora agassizii Studer 1894: 63; Menneking 1905: 253–255, pl. 8, figs 5–6, pl. 9, figs 15–16; Versluys 1906: 112; Kükenthal 1919: 475; 1924: 317; Cairns 2009: 422–424 (key and phylogenetic analysis); Cairns and Bayer 2009: 31 (listed).

######## Material examined.

*JSL-I*-1922, 1 colony, USNM 1297148. *JSL-I*-1931, 3 branches, USNM 1161747; *JSL-I*-1935, 1 branch, USNM 1161749; *JSL-II*-3108, 1 colony, USNM 1093041; *JSL-I*-3907, 1 colony, USNM 1297151; syntypes.

######## Types.

Syntypes: several branches from which 99% of the polyps are detached, MCZ 4815, and SEM 1357–1358, NMNH. Mixed in with the type lot of *C. agassizii* were several branches of *Narella ambigua*, a species collected from the previous *Albatross* station (3403). These specimens were separated by me in 2008.

######## Type locality.

*Alb*-3404: 1°03'S, 89°28'W (south of San Cristóbal, Galápagos), 704 m depth.

######## Distribution.

Galápagos: west of Isabela, off Marchena and San Cristóbal, 509–1545 m deep.

######## Description.

The colony is uniplanar, equally and dichotomously branched; the largest colony (*JSL-I*-3108, Figure 2c) is 13 cm tall and 10 cm in width, with a basal axis diameter of 1.1 mm. The colonies are quite flexible and weak; the polyps are poorly attached and often detached after collection. Polyps are directed downward, and occur in whorls of four to six, the whorl diameter measuring 3.5–4.5 mm; the whorls are closely spaced, 4–4.5 whorls occurring per cm branch length. The horizontal length of the polyp is 1.8–2.2 mm. The pale yellow axis is slender, contributing to the flexibility of the colony.

**Figure 7.**
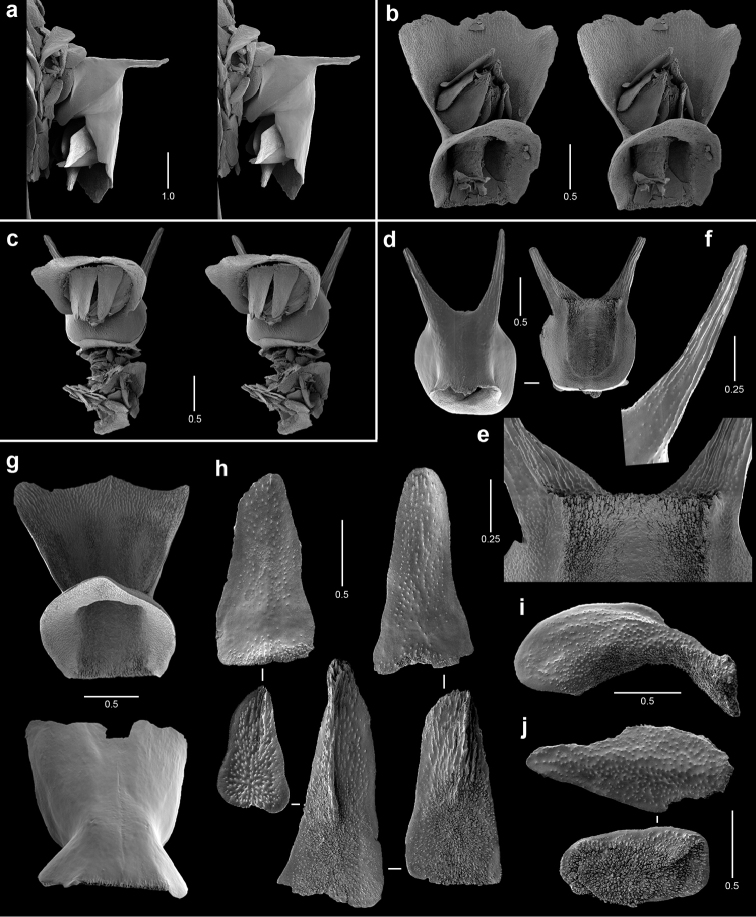
Polyps and sclerites of *Calyptrophora agassizii* from the holotype, *Alb*-3404 , MCZ 4815. **a** lateral stereo view of a polyp **b–c** adaxial stereo views of a polyp **d** basal scales **e** articulating ridge of inner basal scale **f** longitudinal ridging on a basal spine **g** two buccal scales **h** opercular scales **i** infrabasal scales **j** coenenchymal scales.

The fused basal scale (Figures 7d–f) stands perpendicular to the branch and is up to 2.1 mm in height, the distalmost 0.9–1.1 mm consisting of two elongate spines (Figure 7 d, f). These spines are broad basally but attenuate to projections that are circular in cross section and bear low longitudinal ridges on all their surfaces (Figure 7f). The outer surface of the basal scale is smooth (Figure 7d); the articulating ridge (Figure 7e) is well developed, about 0.7 mm in length. The fused buccal scale (Figure 7g) is up to 2.2 mm in length and is oriented parallel to the branch. The distal edge of the buccal scale consists of several broad lobes (not spines or teeth), which form a translucent cowl (Figure 7b, c) up to 0.8 mm in length, which encircles and protects most of the operculum. Like the basal scales, the outer surface of the buccal scales is smooth, the inner face covered with low granules arranged in longitudinal rows near the distal edges. The paired, strongly curved infrabasals (Figure 7i) are about 1 mm in width and only 0.3 mm in height. The opercular scales (Figure 7h) range in length from 0.45–0.90 mm, decreasing in length from ab- to adaxial polyp side, forming a relatively small operculum that is encircled by the buccal cowl; their L:W ranges from 1.9–3.3. The operculars are thin, flat, and triangular, with a blunt apex; their outer surface is smooth to slightly granular, their inner surface covered by a longitudinal keel. The coenenchymal scales (Figure 7j) are thin, flat, and elongate, with blunt ends, and up to 0.8 mm in length. Their outer surface is covered with small, low granules.

**Comparisons**. Only four of the 23 species of *Calyptrophora* have polyps oriented in the downward direction (Cairns 2009). *Calyptrophora agassizii* easily differentiated from the others by having equal, dichotomous branching and keeled opercular scales.

**Remarks**. Several monographers have redescribed this species, but apparently based only on the type material. This is the first subsequent report of the species based on new material. The latitude reported by Studer (1894) for the type specimens is incorrect, it being 1°03'S, not north latitude.

####### Calyptrophora reedi sp. n.

Taxon classificationAnimaliaAlcyonaceaPrimnoidae

http://zoobank.org/FEE055B3-EE29-470E-9398-41A48442B5C0

Figures 2d , 8

######## Material examined.

**Types**. Holotype: colony and SEM stubs 2334–2337, *JSL-I*-3930, USNM 1409027. Paratype: *JSL-I*-1922, 1 denuded colony with many detached polyps, USNM 1409028; *Nautilus* NA-064-126-01-A, 1 branch, CDRS.

######## Type locality.

*JSL-I*-3930: 0°29.755'S, 90°13.98'W (northeast coast of Santa Cruz, Galápagos), 450 m depth.

######## Distribution.

Galápagos: off Santa Cruz, Santiago, and Marchena, 445–509 m depth.

######## Description.

**Figure 8.**
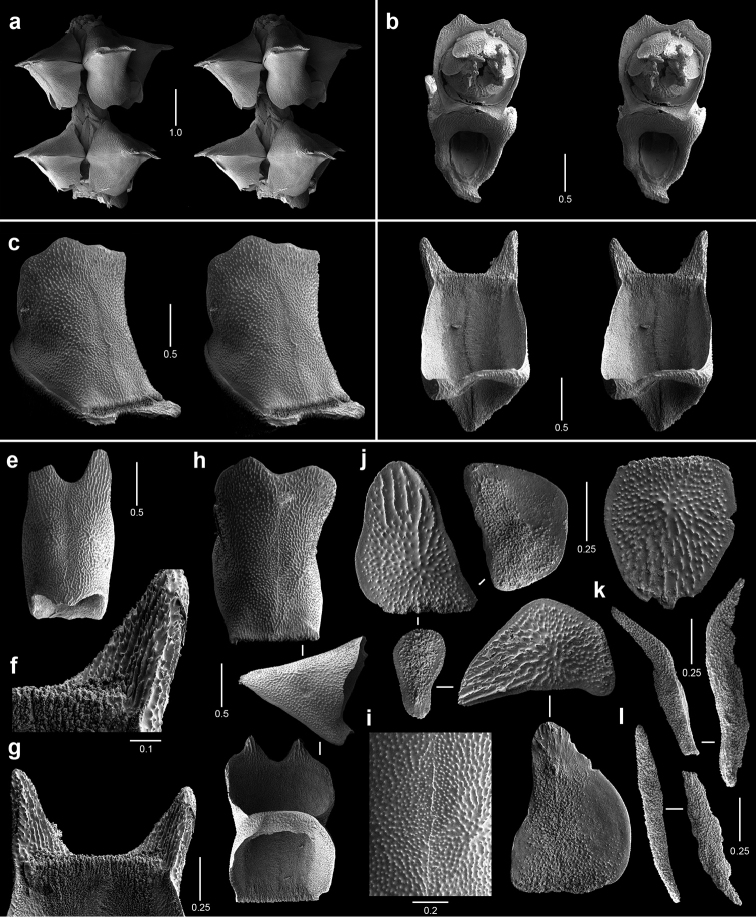
Polyps and sclerites of *Calyptrophora reedi* from the holotype, USNM 1409027. **a** lateral stereo view of two whorls **b** adaxial stereo view of a polyp **c** buccal stereo view of a polyp **d** stereo view of inner face of a basal scale **e** basal scale **f** ridged inner spine of basal scale **g** articulating ridge and spines of a basal scale **h** buccal scales **i** abaxial fusion of buccal scales **j** six opercular scales **k** two infrabasal scales **l** two coenenchymal scales.

The colony is uniplanar, equally and sparsely dichotomously branched, some end branches up to 12 cm in length. The holotype (Figure 2d) is only 6.6 cm in height, whereas the paratype from *JSL-I*-1922 is larger (20 cm) but poorly preserved. Polyps are directed downward, and occur in whorls of four to six (Figure 8a), the whorl diameter measuring 4.0–5.5 mm; the whorls are closely spaced, about four polyps occurring per cm branch length. The horizontal length of a polyp is 2.2–2.4 mm. The axis is dark brown to bronze color.

The fused basal scale (Figures 8a, d, e) stands at roughly a 45° angle to the branch, and is 2.0–2.3 mm in height, including its distal spines. The distal spines (Figure 8d, f,) are flattened, with a broad base 0.30–0.35 mm in width, and extend 0.45–0.55 mm above the articulating ridge. Both inner and outer surfaces of the basal spines are covered with 6–11 thin, parallel spinose ridges. The straight articulating ridge is well defined (Figure 8d, f, g) and 0.70–0.75 mm in length. The outer surface of both basal and buccal scales is covered homogeneously with small but sharp spines. The fused buccal scale (Figure 8c, h) is 1.5–1.9 mm in length and slopes downward toward the branch surface, making the articular ridge and basal spines by far the highest point of the polyp. The distal edge of the buccal scale is smooth and usually bilobate, although the distal edges of some polyps are produced into two blunt teeth up to 0.3 mm in height (Figure 8h, lower figure); the buccal distal edge forms a cowl (Figure 8b) that completely enveloped the operculum. A low mid-dorsal (sagittal) ridge (Figure 8i) is often seen as a remnant of the dorsal fusion of the two young buccal scales. The abaxial opercular scale (Fig. 8j, upper right) is almost elliptical in shape, up to 0.62 mm in height, and has a L:W as low as 1.15. The adaxial opercular (Figure 8j, lower left) is much smaller (0.35–0.45 mm in length) and triangular, having a L:W of about 1.7. The lateral operculars are asymmetrical, each having a lateral shoulder on their adaxial side. They range from 0.58–0.69 mm in height and have a L:W of 1.4–1.7. All opercular scales are quite thin and delicate, their outer surface covered with small spines often aligned as low ridges distally; their inner surface is tuberculate and not keeled (Figure 8j). The coenenchymal scales (Figure 8l) are straight, thick, and elongate, up to 1.1 mm in length and about 0.12 mm in width, with a L:W up to 10. They bear surface ornamentation like that of the body wall scales. Four thick, elongate coenenchymal scales (Figure 8k) surround the base of each polyp, each up to 1.2 mm in length with a L:W up to 6, and slightly curved to conform to the curvature of the polyp attachment of the branch, thus functioning as infrabasals.

######## Comparisons.

Only four species in the genus have downward oriented polyps, the so called *wyvillei*-complex sensu Bayer (2001), *C. reedi* being very similar to *C. agassizii*, also known from the Galápagos. *Calyptrophora reedi* differs from that species primarily in the shape of its basal scales, which are not as long as those of *C. agassizii*, have a broader base, and a non-pointed (flat) tip.

######## Etymology.

Named in honor of John Reed (HBOI), who participated in the *JSL-I* Galápagos expedition of 1986, during which this species was collected.

####### Genus Narella

Taxon classificationAnimaliaAlcyonaceaPrimnoidae

Gray, 1870

Narella Gray 1870: 49; Cairns and Bayer 2003: 618–619; 2007b: 84–86; Cairns and Bayer 2009: 43; Cairns and Baco 2007: 392–393; Cairns 2012: 14.
Stachyodes Wright and Studer 1889: 49; Versluys 1906: 86–88; Kükenthal 1919: 452–456.

######## Type species.

*Primnoa regularis* Duchassaing and Michelotti, 1860, by monotypy.

######## Diagnosis.

(from Cairns 2012): Colonies dichotomously branched (laterally), pinnate, or unbranched. Polyps arranged in whorls, all polyps facing downward. Each polyp covered with three (rarely four) pairs of abaxial body wall scales, one pair of basals, one or two pairs of medials, one pair of buccals, and one to four pairs of small adaxial scales, nevertheless leaving the adaxial side largely naked. Articular ridge present between basal and medial body wall scale. Distal margins of body wall scales often spinose, toothed, or lobate, often extending as a protective cowl. Opercular scale usually keeled. Coenenchymal scales often quite thick, of variable shape, sometimes ridged (i.e., “sail-scales”). Tentacular platelets often present.

######## Distribution.

All ocean basins except Arctic, 128–4594 m deep.

######## Remarks.

Previously (Cairns 2012) I stated that there were 43 valid species of *Narella*, but I inadvertently counted two junior synonyms within this tabulation, so, in fact, there are only 41 valid species. The new species described herein brings the total to 42. A unified key to all species known after 1924 (Kükenthal 1924) does not exist, but regional keys are available for 29 of these species: the seven western Atlantic species (Cairns and Bayer 2003), the nine Hawaiian species (Cairns and Bayer 2007b), the five Alaskan species (Cairns and Baco 2007), and the eight New Zealand species (Cairns 2012).

####### Narella ambigua

Taxon classificationAnimaliaAlcyonaceaPrimnoidae

(Studer, 1894)

Figures 2e , 9

Stachyodes ambigua Studer 1894: 63–64; Menneking 1905: 248–251, pl. 8, figs 1–2, pl. 9, figs 11–12; Versluys 1906: 103–104; Kükenthal 1919: 464 (key to species); 1924: 314.
Narella ambigua Cairns and Bayer 2007 (not 2008): 86 (listed); Cairns 2007b: 512 (listed); Cairns and Bayer 2009: 30 (listed).

######## Material examined.

Branch fragments and detached polyps from *Alb*-3404, MCZ 79048, and USNM 1405230 (topotypic: possible syntypes); *Alb*-2818, 1 colony and SEM stubs 2312–2315, USNM 44165; *Gilliss*-21, 1 branch, USNM 57576; *JSL-I*-1927, 1 colony, USNM 1297223.

######## Types.

As mentioned in the account of *Calyptrophora agassizii*, about 65% of the type lot (branches and detached polyps) of that species consisted of *Narella ambigua*. *Narella ambigua* was collected at the previous station (*Alb*-3403) to that of *C. agassizii* (*Alb*-3404), approximately 20 km to the northeast and bathymetrically 2 m shallower, both stations from off the southern coast of San Cristóbal. The *Narella* specimens were separated from the type lot of *C. agassizii* in 2008 and cataloged as MCZ 79048. Since the type of *S. ambigua* could not be found at the MCZ in 2008, the specimens cataloged as MCZ 79048 may serve as representative topotypic specimens, and may in fact be type material. A fragment of this colony is also deposited at the NMNH (USNM 1405230).

######## Type locality.

*Alb*-3403: 0°58'30"S, 89°17'W (south of San Cristóbal, Galápagos), 702 m depth.

######## Distribution.

Galápagos: off Santiago, Santa Cruz, and San Cristóbal), 702–741 m deep. Elsewhere: off Panama, 1463 m depth (herein, *GS*-21).

######## Description.

**Figure 9.**
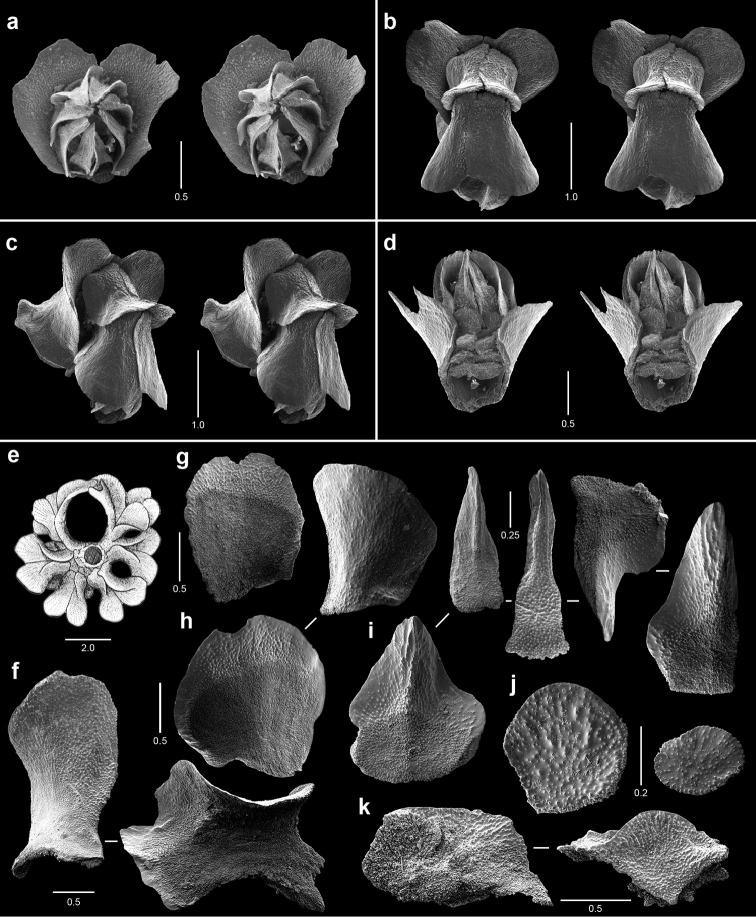
Polyps and sclerites of *Narella ambigua* from *Alb*-2818, USNM 44165. **a** opercular stereo view of a polyp **b–d** abaxial, lateral, and adaxial stereo views of a polyp, respectively **e** axial view (drawing) of a whorl showing polychaete tube **f** basal scales **g** medial scale **h** buccal scales **i** opercular scales **j** adaxial buccal scales **k** coenenchymal scales.

The colony is uniplanar, and dichotomously (laterally) and sparsely branched (Figure 2e), large colonies being up to 28 cm in height and up to 1 cm in basal branch diameter. Terminal branches may be quite long, up to 15 cm. The axis is pale yellow. The polyps are arranged in whorls of five to seven (Figure 9e); whorls are not directly adjacent to one another and thus there are only approximately 2.5 whorls per cm branch length; the whorl diameter of terminal branchlets is about 6–7 mm. The horizontal length of a polyp is 2.5–3.0 mm.

The basal scales (Figure 9c, f) stand perpendicular to the branch and extend up to 2.8 mm in height, the distal 0.6–0.7 mm portion projecting beyond the junction with the medial scales as a broad lobate extension (Figure 9b). The lateral edge of one of the basal scales of a polyp will often enlarge and curve toward the corresponding enlarged basal scale of the adjacent polyp, forming a solid tube up to 3.5 mm in diameter that houses a commensal polychaete worm (Figure 9e). The dorso- and anterolateral faces of the basal scales are gently curved, not ridged. The medial scales (Figure 9g) are narrow, 0.9–1.1 mm in length, and have upturned edges proximally and distally (saddle-shaped). The buccal scales (Figure 9b, c, h) are longer (up to 1.6 mm) and about twice as wide as the medials, their distal edges rounded and smooth, forming a cowl (Figure 9a) up to 0.6 mm that encircles the operculum; the distal edges of the two buccal scales form a bilobate shape for the tip of each polyp, not unlike the distal edges of the basal scales. The ratio of the major body wall scales is about: 1:0.6:0.7. There are four pairs of small elliptical adaxial body wall scales (Figure 9d, j), ranging from 0.26 to 0.42 mm in greater diameter. The outer faces of all body wall scales are covered with small granules and thus look rather smooth. All of the opercular scales (Figure 9i) are roughly the same length, ranging from 1.0–1.3 mm in length, but the single abaxial opercular is quite broad (e.g., L:W = 1.2), whereas the single adaxial is quite slender (e.g., L:W = 3.0). The other six lateral operculars usually have a basal shoulder on their adaxial edges and thus have an intermediate L:W ratio. The outer surface of the operculars is granular like the body wall scales, whereas the inner surface bears a rounded keel. The coenenchymal scales (Figure 9k) are irregular to polygonal in shape, up to 1.4 mm in length, and have a flat to slightly concave outer surface.

######## Comparisons.

*Narella ambigua* is easily distinguished from the somewhat similar *Paracalyptrophora enigma* by its long terminal branches, polychaete commensalism that causes highly modified basal scales, fewer polyp whorls per cm, non-toothed basal scales, lack of an articular ridge, and granular (not ridged) coenenchymal scales.

######## Remarks.

Although discussed by several authors through the years (see synonymy), this is the first subsequent report of this species since its original description.

####### Genus Paracalyptrophora

Taxon classificationAnimaliaAlcyonaceaPrimnoidae

Kinoshita, 1908

######## Type species.

*Calyptrophora kerberti* Versluys, 1906, by subsequent designation (Cairns and Bayer 2004).

######## Diagnosis.

Colonies dichotomously branched in one plane, sometimes in a lyrate pattern and sometimes as two parallel fans. Polyps arranged in whorls of up to eight, all polyps pointed downward. Each polyp covered with two (rarely three) unfused pairs of body wall scales. Articluar ridge present between basal and buccal (or medial) body wall scales. Distal margins of buccal scales smooth or spinose. A pair of infrabasal scales often present. Coenenchymal scales elongate, granular, and sometimes ridged.

######## Distribution.

Southwestern Pacific Ocean, Galapagos, Japan, Hawaii, North Atlantic, 150–1480 m deep.

######## Remarks.

The genus diagnosis is herein expanded to accommodate a species having three pairs of body wall scales, otherwise similar to the genus *Narella*. The character placing it in *Paracalyptrophora* is its possession of an articular ridge, which is found in this genus as well as *Calyptrophora*, and is considered more significant than the number of body wall scales.

####### Paracalyptrophora enigma sp. n.

Taxon classificationAnimaliaAlcyonaceaPrimnoidae

http://zoobank.org/14AC2E7D-3E1F-4701-92AD-F8B152150ADB

Figures 3a , 10

######## Material examined.

**Types**. Holotype: colony and SEM stubs 2338–2342, *JSL-I*-1915, USNM 1409703. Paratype: *JSL-I*-1916, 1 colony, USNM 1409707.

######## Type locality.

1°17.2'S, 89°48.7'W (northwest of Española, Galápagos), 653 m depth.

######## Distribution.

Known only from northwest of Española, Galápagos, 547–653 m deep.

######## Description.

**Figure 10.**
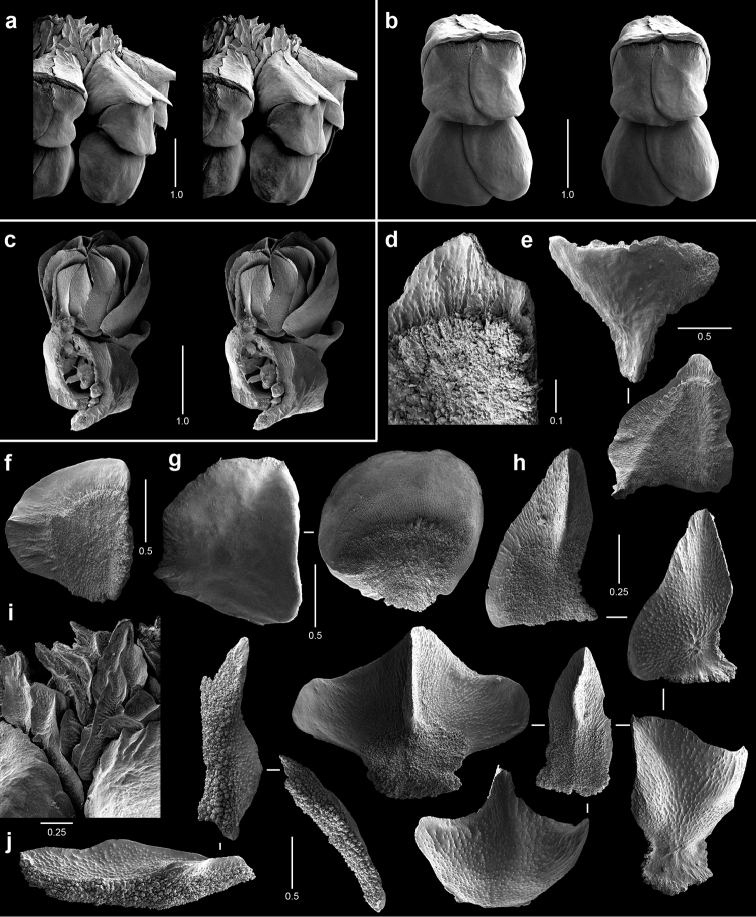
Polyps and sclerites of *Narella enigma* from the holotype, *JSL-I*-1915, USNM 1409703. **a** lateral stereo view of a whorl **b–c** abaxial and adaxial stereo views of a polyp, respectively **d** articular ridge and inner ridging on a basal spine **e** basal scales **f** medial scale **g** buccal scales **h** opercular scales **i** coenenchymal scales, in situ **j** coenenchymal scales.

The colony is uniplanar, equally and dichotomously branched, the largest colony (the holotype, Figure 3a) being 17 cm in length and having only 12 terminal branches, none longer than 4 cm. Its broken base is highly calcified and 10.5 mm in diameter. The entire corallum is white. The polyps are arranged in closely spaced (4.5–5 polyps per cm) whorls of seven or eight (Figure 10a), the higher number occurring on larger-diameter basal branches; the whorl diameter ranges from 4.5–6.0 mm. The horizontal length of a polyp is 2.5–2.7 mm.

The basal scales (Figures 10a, e) stand perpendicular to the branch or tilted slightly anteriorly, and extend up to 1.75 mm in length, the distal 0.23–0.28 mm of each basal scale projecting as wide, flat teeth, which are longitudinally ridged on their inner surface (Figure 10d). There is a horizontal articular ridge joining the basal to the medial scales (Figure 10d). Otherwise, the inner surface of the basal scale is highly tuberculate, and its outer surface is smooth and not ridged, as are all the body wall scales. The medial scales (Figure 10f) are as wide as the buccals, 1.10–1.15 mm in length; the distal and lateral 0.25 mm of their inner surface is smooth (but not ridged), the rest highly tuberculate. The buccal scales (Figure 10g) are slightly longer (1.12–1.25 mm) but much wider, curved around much of the operculum in a scalloped shape. The 0.5 mm distal, inner margins of these scales are also smooth and thin, forming a translucent cowl (Figure 10c) surrounding the operculum. The ratio of the major body wall scales is about: 1:0.67:0.77. There is at least one pair of rectangular adaxial scales (Figure 10c), each about 0.37 mm in width. The single abaxial opercular scale (Figure 10h, leftmost) is 0.85–0.90 mm in length and has two broad lateral lobes (producing a very low L:W of 0.75–0.85) and thus being symmetrical, and has a small rectangular base. The much smaller, symmetrical, paired adaxial operculars are 0.75–0.80 mm in length with a L:W of about 2.0. The five lateral operculars range in size from 0.90 to 1.05 mm in length and are asymmetrical, each having a lobe on their adaxial side, the L:W ranging from 1.5–1.6 (Figure 10h). The distal inner half of all opercular scales bears a thin ridged keel, whereas the outer surface is covered with low pointed granules. The coenenchymal scales (Figure 10i, j) are elongate (L:W up to 8) and longitudinally crested (i.e., “sail scales”, Figure 10j), the crests up to 0.15 mm in height. The outer surface of these scales is covered with small granules much like that of the operculars.

######## Comparisons.

Superficially this species resembles the genus *Narella*, in that it has three pairs of body wall scales, but the distal inner surface of the basal scales have an articular ridge, which is more consistent with the genus *Paracalyptrophora*, *P. enigma* being the only species in the genus with three pairs of body wall scales.

######## Etymology.

Named “*enigma*” (Latin for inexplicable) because it is the only species in the genus to have three (not two) pairs of body wall scales.

####### Genus Plumarella

Taxon classificationAnimaliaAlcyonaceaPrimnoidae

Gray, 1870

Plumarella Gray 1870: 36; Kükenthal 1919: 340–343; Bayer 1981: 936 (key to genus); Cairns and Bayer 2009: 39–40; Cairns 2011: 7; 2016: 51–52.

######## Type species.

*Gorgonia penna* Lamarck, 1815, by subsequent designation (Wright and Studer 1889).

######## Diagnosis.

Colonies usually uniplanar and alternately pinnately branched, dichotomously branched, or bottlebrush in shape. Polyps arranged in alternate biserial fashion (nominate subgenus), crowded on all sides (subgenus Dicholaphis), paired (subgenus Faxiella), or arranged in whorls (subgenus Verticillata). Each polyp covered by eight rows of body wall scales, the adaxial scales usually somewhat smaller. Distal edges of marginal body wall scales do not overlap much of opercular scales. Inner surface of opercular scales may be smooth or ridged, but not keeled, except in subgenus Faxiella.

######## Distribution.

Indo-Pacific, western Atlantic, Subantarctic, 10–3181 m deep.

######## Remarks.

There are 37 species in the genus arranged in four subgenera, most listed in Cairns (2011: table 2), however, two new subgenera were recently added by Zapata-Guardiola and López-González (2012) and Zapata-Guardiola, López-González and Gili (2012).

####### Plumarella (Faxiella) abietina

Taxon classificationAnimaliaAlcyonaceaPrimnoidae

(Studer, 1894)

Figures 3b , 11

Amphilaphis abietina Studer 1894: 65; Menneking 1905: 255–260, pl. 8, figs 7–8, pl. 9, figs 17–20; Versluys 1906: 22; Cairns 2007b: 512 (listed); Cairns and Bayer 2009: 28 (listed): Cairns 2016: 58.
Thouarella (Amphilaphis) abietina Kükenthal 1919: 410–411; 1924: 290.
Not Thouarella abietinaPasternak 1981: 49–50 (=T. vityazi Zapata-Guardiola and López-González 2012).
Plumarella (Faxiella) abietina Zapata-Guardiola and López-González 2012: 372–375, figs 20–22.

######## Material examined.

*Alb*-2818, 1 colony in very poor condition (most of its polyps detached) and SEM stubs 2343–2346, USNM 49605; *JSL-I*-1929, 1 branch, USNM 1406397; holotype.

######## Types.

Holotype: *Alb*-3399, MCZ 4802.

######## Type locality.

1°07'N, 81°04'W (lower continental slope off northwestern Ecuador, at same latitude as Galápagos Islands but about 860 km to the east), 3181 m depth.

######## Distribution.

Galápagos: east of Santa Cruz and off Roca Redonda, 717–808 m deep. Elsewhere: off Ecuador, 3181 m depth.

######## Description.

The colony is uniplanar to slightly bushy, the Galápagos specimen (*Alb*-2818, Figure 3b) measuring 12 cm in height, but it is part of a badly damaged colony that was probably larger. Branching is equal and dichotomous. The upward-directed polyps are usually arranged in pairs but may also occur in whorls of three as well as individually. Polyps are about 2.6 mm in length, about two pairs occurring per cm branch length.

**Figure 11.**
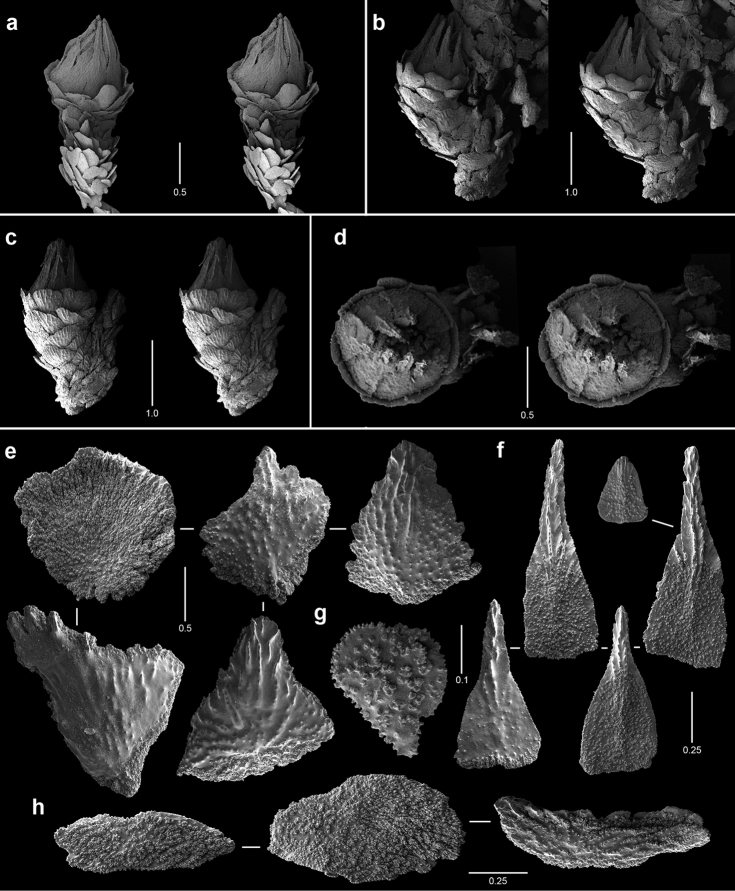
Polyps and sclerites of *Plumarella abietina* (**a** holotype, *Alb*-3399, MCZ 4802 **b–h** *Alb*-2818, USNM 49605) **a–d** abaxial, lateral, abaxial, and opercular stereo views of a polyp **e** body wall scales **f** opercular scales **g** adaxial scale **h** coenenchymal scales.

The body wall scales (Figure 11e) are arranged in eight somewhat irregular longitudinal rows, the sclerite formula being: 5–6:4–5:4–5: variable. All marginal scales, except for the abaxial marginals, are roughly rectangular, 0.35–0.64 mm in width, and have a straight distal margin that covers only a small proximal part of the opercular scales. The distal edges of the two marginal scales project as small teeth. All body wall scales except for the adaxials become progressively larger and transform to triangular toward the base of the polyp. The adaxial body wall scales (Figure 11a, g) are small (0.25–0.35 mm in diameter) and random (variable) in arrangement, but cover the entire adaxial side of the polyp. The body wall scales are relatively thick, convex, and covered with low ridges; their distal edges are finely serrate. The opercular scales (Figure 11f) are triangular in shape (0.75–1.2 mm in length, L:W = 2.2–2.9), each with an elongate distal process, the length and L:W progressively decreasing from ab- to adaxial side; the operculum is quite prominent (Figure 11a–c). The outer opercular face is covered with granules basally and short serrate ridges distally; the inner face is also covered with serrate ridges raised into a keel-like structure distally, but having tubercles proximally. The coenenchymal scales (Figure 11h) are elongate (L:W about 4), with a granular outer surface and a tuberculate inner surface.

######## Comparisons.

This is the deepest of the 37 known species in the genus. Only one other species occurs in this subgenus, P. (F.) delicatula (Thompson and Rennet, 1931), known only from the New Zealand region at 650–2743 m depth (Cairns 2016). These species are compared by Cairns (2016).

######## Remarks.

The specimens reported herein are only the second report of the species. It fits the re-description of the holotype given by Zapata-Guardiola and López-González (2012), except that the Galápagos specimen sometimes has polyps arranged in whorls of three.

####### Genus Parastenella

Taxon classificationAnimaliaAlcyonaceaPrimnoidae

Versluys, 1906

Stenella Wright and Studer 1889: 56 (junior homonym).
Stenella (Parastenella) Versluys 1906: 39, 45.
Parastenella Bayer 1961: 295; 1981: 936; Cairns 2007a: 245–247; 2007b: 518; Cairns and Bayer 2009: 45–46, figs 16A–G; Cairns 2010: 434; 2016: 94–96.

######## Type species.

*Stenella doderleini* Wright and Studer, 1889, by subsequent designation (Bayer 1956).

######## Diagnosis.

Colonies uniplanar to slightly bushy; branching lateral and somewhat irregular. Polyps stand perpendicular to branch, arranged independently or in pairs or whorls of up to four. Eight marginal scales present, offset in position from opercular scales; marginal, and sometimes submarginal, scales fluted; nematocyst pads present on distal inner surface of fluted marginals. Body wall scales arranged in four to eight longitudinal rows. Operculum well developed, the distal inner surface of operculars prominently keeled. Coenenchymal scales flat to highly concave, sometimes ridged. Pinnular rodlets sometimes present.

######## Distribution.

Cosmopolitan, except for eastern Atlantic, the Arctic, and off continental Antarctica, 475–3470 m depth.

######## Remarks.

Including the new species described herein, there are eight species known in this distinctive genus. Accounts of *Parastenella* species are found in Cairns (2007b, 2010, 2016) and Cairns and Bayer (2009).

####### Parastenella pomponiae sp. n.

Taxon classificationAnimaliaAlcyonaceaPrimnoidae

http://zoobank.org/3E4C0441-A172-4E29-8F5A-8CA4E035B7A1

Figures 3c , 12

######## Material examined.

**Types**. Holotype: colony and SEM stubs 2303–2305, *JSL-I*-1922, USNM 1410289. Paratypes: *JSL-I*-1922, 1 colony, USNM 1410290; *JSL-I*-3912, 1 branch, USNM 1410291; *Nautilus* NA064-125-01-A, 1 colony, CDRS.

######## Type locality.

0°23.68'N, 90°26.341'W (off northeastern Marchena), 475–578 m deep.

######## Distribution.

Galápagos: off Marchena, Santiago, and Isabela, 446–578 m deep.

######## Description.

**Figure 12.**
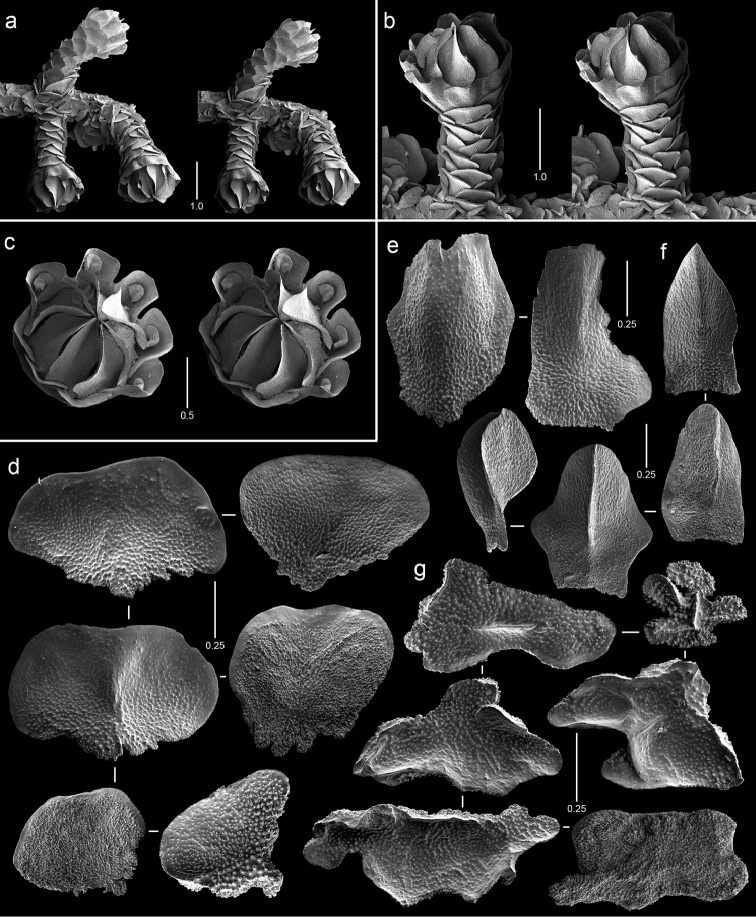
Polyps and sclerites of *Parastenella pomponiae* from the holotype, *JSL-I*-1922, USNM 1410289, **a** stereo view of paired polyps **b** lateral stereo view of a polyp **c** opercular stereo view of a polyp showing nematocyst pads **d** body wall scales **e** fluted marginal scales **f** opercular scales **g** coenenchymal scales.

The colony is uniplanar, the largest colony (the holotype, Figure 3c) measuring 44 cm in height, and having a basal branch diameter of 6.5 mm. Branching is lateral and somewhat irregular; the longest terminal branchlets are less than 2 cm in length. The axis is golden bronze and the polyps and coenenchyme are white. The polyps are 2.8–3.1 mm in height and stand perpendicular to the branches, arranged in pairs (Figures 12a, b), whorls of three, and often as singles; about five polyp whorls (or pairs) occur per cm branch length.

The body wall scales (Figure 12d) are arranged in eight rows, each row with six scales, the lateral edges of all scales overlapping with those of adjacent rows. The eight marginal scales form an asymmetrical rosette: six of the marginals consist of elongate scales (up to 0.9 mm in length and 0.4 mm wide) with broad shallow flutes, whereas the two adaxial marginals are much wider but shorter (up to 0.6 in length and 0.9 mm in width) and are flat (without a flute) or bear only a very shallow flute (Figure 11c). A nematocyst pad (Figure 12c) is present on the distal inner face of each fluted marginal. The body wall scales proximal to the marginals (Figure 12b) are never fluted and are elliptical in shape, about 0.60–0.85 mm in width; they have a concave granular outer surface, their lateral and distal edges turned upward. The opercular scales (Figures 12b, c, f) are fairly uniform in length (0.7–0.8 mm) but variable in width. The abaxial and adaxial operculars are symmetrical, the abaxial having two lateral lobes (H:W about 1.2), the adaxials lacking lobes (H:W about 2); the lateral operculars are asymmetrical, each having only one lobe on the adaxial lateral side. All opercular scales have a deeply longitudinally creased outer surface that corresponds to a sharply keeled inner surface. The coenenchymal scales (Figure 12g) are irregular in shape, although usually longer than broad, and up to 1.1 mm in length. Their outer surface is concave, like that of the body wall scales, and bears low granules and occasionally short ridges.

######## Comparisons.

*Parastenella pomponiae* is morphologically most similar to *P. ramosa* (Studer, 1894), known from the eastern Pacific from the Gulf of Alaska to Panama, but differs from that species in having eight (not five) rows of body wall scales, concave (not flat) coenenchymal scales, wider fluted marginal scales, and in lacking submarginal fluted body wall scales.

######## Etymology.

Named in honor of Shirley Pomponi (formerly of HBOI), who participated in the *JSL-I* expedition of 1986, during which this species was collected.

####### Genus Chrysogorgia

Taxon classificationAnimaliaAlcyonaceaPrimnoidae

Duchassaing and Michelotti, 1864

Chrysogorgia Duchassaing and Michelotti 1864: 13; Versluys 1902: 17–33; Kükenthal 1919: 505–511 (key to species); 1924: 388–390 (key to species); Bayer 1956: F216; Bayer and Stefani 1988: 259 (key to genus and some species); Cairns 2001: 754–756; Pante and France 2010: 600 (key to genus); Pante et al. 2012: fig. 2.
Dasygorgia Verrill 1883: 21.

######## Type species.

*Chrysogorgia desbonni* Duchassaing and Michelotti, 1864, by monotypy.

######## Diagnosis.

Branching from main branch sympodial in an ascending spiral, clockwise (R) or counterclockwise (L), usually following a repeated geometric branching formula, producing a bottlebrush colony, or dichotomous in one or more parallel planes. Branchlets repeatedly dichotomously branched, resulting in short terminal segments. Polyps large in relation to branchlets, standing perpendicular to branchlets and usually well separated. Sclerites consist of rods and scales. Axis with a brilliant metallic luster, usually golden or yellow in color, and thus referred to as the golden corals.

######## Distribution.

Cosmopolitan, including off Antarctica (Pante et al. 2012: figs 4–5), 100–3860 m deep.

######## Remarks.

In order to manage the relatively large number of species in the genus, now standing at 70, Versluys (1902) divided the genus into three group based on the presence of rods or scales in the body wall and tentacles of each species, which he referred to as Groups A-C. Table 1 is a graphic representation of the four permutations of these two characters as divided between the two regions of the polyp. This table also lists the number of species currently assigned to each group, their geographic and depth ranges, and the branching formulas encountered within the group. A species having the fourth permutation, Group D, was not reported until 2015 (Cordeiro et al. 2015). Recent molecular evidence (Pante et al. 2012), based on three genes, supports the monophyly of the genus as well as groups B and C, but results in Group A being paraphyletic. Regardless of the true phylogeny of the species, the grouping of Versluys (1902), along with the branching formula, served to help distinguish the various species.

**Table 1.** The four species groups of Chrysogorgia determined by a combination of body wall and tentacular sclerite type.

	Body Wall Rods	Body Wall Scales
Tentacular Rods	Group A: 38 species: Indo-West Pacific, Atlantic; 100–3114 m. 2/5R, 1/4L, 3/8L, 1/3R, 2/5L, 1/3L, dichotomous, irregular, pinnate	Group B: 13 species: Indo-West Pacific, western Atlantic; 250–2271 m. 1/4R, 1/5R, 1/6R, 1/7R, 1/7R, 2/5R, biflabellate
Tentacular Scales	Group D: 1 species: off Brazil, 1300 m. dichotomous	Group C: 18 species: Indo-West Pacific, North Atlantic, Antarctic; 204–3860 m. 1/3L, 1/4L, 2/5L, 1/4R, flabellate

####### Chrysogorgia scintillans

Taxon classificationAnimaliaAlcyonaceaPrimnoidae

Bayer and Stefani, 1988

Figures 3d , 13

Chrysogorgia curvata Nutting 1908: 591, pl. 45, fig. 9.
Chrysogorgia scintillans Bayer and Stefani 1988: 270–276, figs 11–12.

######## Material examined.

*JSL-I*-1927, 1 colony and SEM stubs 2327–2331, USNM 89377; *JSL-I*-1942, 1 colony, USNM 1160577; *JSL-I*-3925, 1 colony, USNM 1409700; holotype.

######## Types.

The holotype is deposited at the NMNH (USNM 25371).

######## Type locality.

*Alb*-4153: between Kauai and Moku Manu, 1758–1937 m deep.

######## Distribution.

Galápagos: between Isabela and Santiago; Cocos Island, 628–768 m deep. Elsewhere: Hawaiian Islands, 1758–1937 m deep.

######## Description.

**Figure 13.**
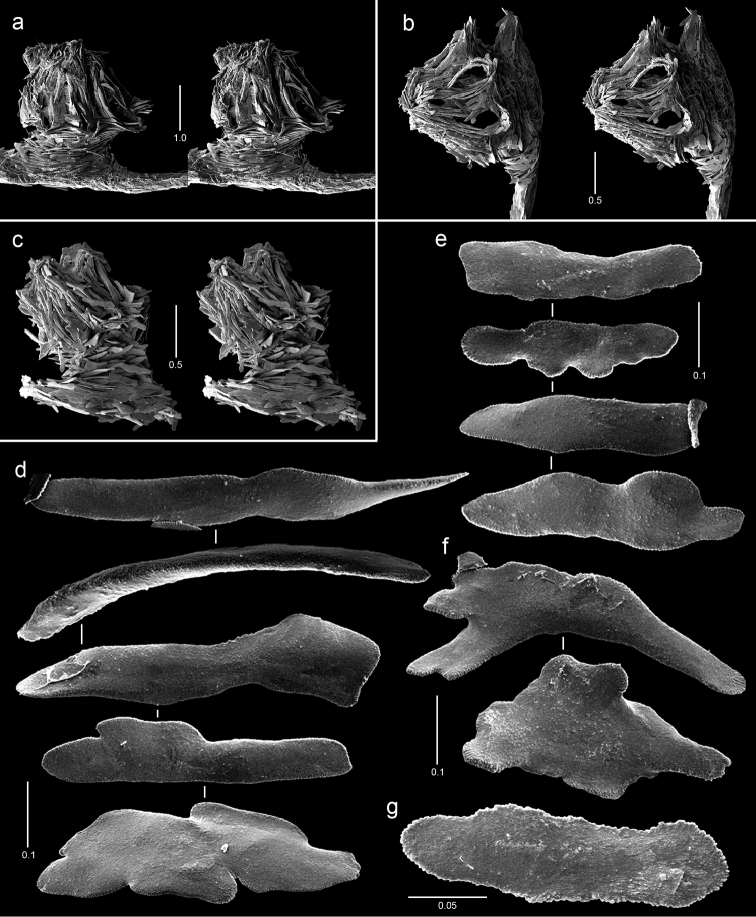
Polyps and sclerites of *Chrysogorgia scintillans* from *JSL-I*-1927, USNM 89377. **a–c** lateral stereo views of polyp showing regions devoid of sclerites **d** body wall scales **e** tentacular scales **f** scales in base of tentacle **g** pinnular scale.

The colony is biplanar (perhaps multiplanar), the largest colony examined (*JSL-I*-1927, Figure 3d) being 25 cm in height and 10 cm in width, and is somewhat bushy. The branching is dichotomous, the length of the internodes 6–7 mm, each internode bearing only one or sometimes two polyps. The polyps are 2.3–2.8 mm in height and project perpendicular to the branches (Figure 13a–c), having a relatively narrow body wall column and a much larger distal crown, made larger by the projecting sclerites that support the tentacles. The axis is a metallic bronze in color.

The body wall scales (Figure 13c, d) are transversely arranged and slightly curved to fit the circumference of the polyp. The body wall scales, called “slippers” by Bayer and Stefani (1988), are up to 0.65 mm in length and 0.07–0.10 mm in width, resulting a L:W ratio ranging from 4.5–6.0; these scales are quite thin (about 0.03 mm). Some body wall scales are often slightly irregular in shape but usually rounded on their distal ends; their inner and outer faces are smooth and their edges rounded. The tentacular scales (Figure 13e) are similar in shape to the body wall scales but somewhat smaller, i.e., 0.35–0.45 mm in length. The base of each tentacle bears a distinctive tear-dropped shaped region about 0.4 mm long and 0.18 mm in width that is devoid of sclerites (Figures 13a-c). These regions are surrounded by flattened scales that project around the lower edge of the tentacle and thus forming a support for it. These scales sometimes have a root-like or lobate base (Figure 13f). The pinnular scales (Figure 13g) are 0.20–0.25 mm in length and are similar to the body wall scales, except that they have slightly serrate marginal edges. The coenenchymal scales (Figure 13c) are indistinguishable from the body wall scales, and are arranged longitudinally parallel to the branch axis.

######## Comparisons.

*Chrysogorgia scintillans* belongs to “Group C” sensu Versluys (1902) (Table 1), i.e., species having sclerites in the form of scales in both tentacles and body wall. Currently there are 18 species known in this grouping (Cairns 2001; Pante and Watling 2011), three of which, including *C. scintillans*, having flabellate or multi-flabellate colonies and non-spiral branching. As noted by Bayer and Stefani (1988: key), *C. scintillans* is most similar to *C. electra* Bayer and Stefani, 1988, another flabellate species, but differs in having larger polyps, smaller projections beneath the tentacle bases, and slightly different shaped to their body wall and coenenchymal scales.

####### Chrysogorgia midas sp. n.

Taxon classificationAnimaliaAlcyonaceaPrimnoidae

http://zoobank.org/62A55FA8-2A1D-429A-8344-B065A33B5696

Figures 3e , 14

######## Material examined.

**Types**. Holotype: colony and SEM stubs 2316–2319, 2350–2316, *JSL-I*-1915, USNM 1160575. Paratypes: *Alb*-2818, many denuded branches (dry), USNM 51464; *JSL-I*-1912, distal colony, USNM 1160579; *JSL-I*-1916, distal colony, USNM 1160578; *JSL-I*-1929, 1 colony, USNM 1160585; *JSL-I*-1933, 1 branch, USNM 1160582; *JSL-I*-3902, 1 branch, USNM 1405908.

######## Type locality.

1°17'12"S, 89°48'42"W (north of Española, Galápagos), 650–662 m deep.

######## Distribution.

Throughout Galápagos from Roca Redondo to Española, 560–816 m deep.

######## Description.

**Figure 14.**
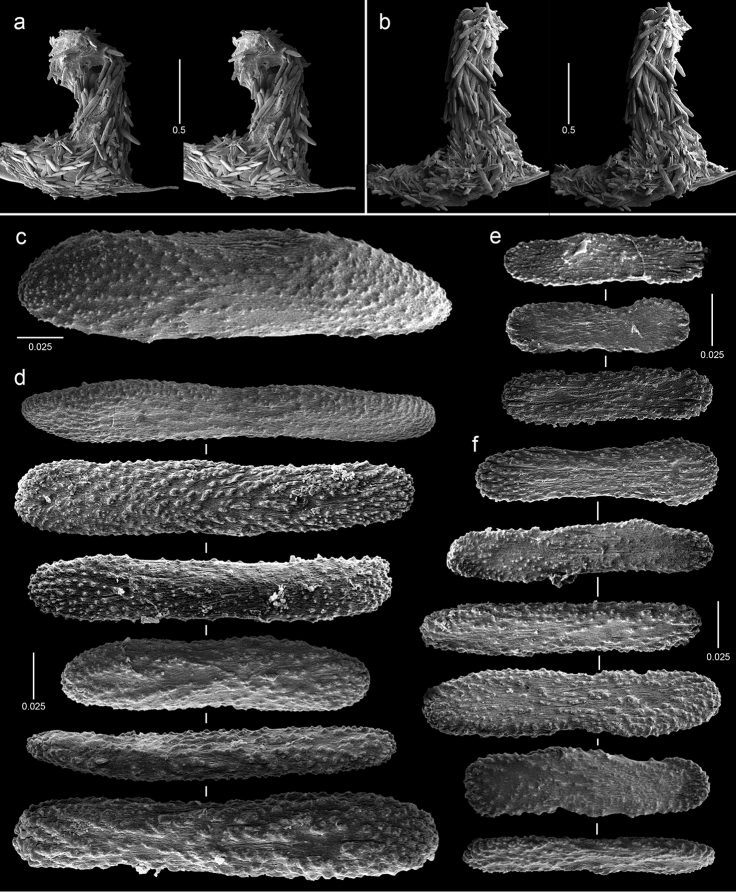
Polyps and sclerites of *Chrysogorgia midas* from the holotype, *JSL-I*-1915, USNM 1160575, **a–b** lateral stereo views of polyps **c** body wall spindle **d** tentacular flattened rods **e** pinnular scales **f** coenenchymal scales.

The colony is bottlebrush in shape (Figure 3e), the holotype measuring 26 cm tall and 12–13 cm in maximum diameter, having a basal branch diameter of 2.5 mm. The branching is sympodial, the branching formula being consistently 1/3L. The orthostiche interval is 12–18 mm. The length of the internodes of the branchlets ranges from 4.0–5.9 mm, up to nine nodes occurring on each branchlet; each internode supports one polyp. The polyps are about 1.1 mm in length, cylindrical (Figures 14a, b), and when preserved in alcohol tend to curve toward the branch surface, the tentacles often adhering to the surface branch. The axis is bronze in color.

The body wall sclerites (Figure 14a–c) are slightly flattened, rotund rods 0.22–0.25 mm in length, having a L:W of 5–6. They are straight and longitudinally oriented. The tentacular sclerites (Figure 14d) are similarly shaped rods, but are slightly shorter (0.18–0.22 mm in length) and more elongate (L:W = 5–8), also longitudinally oriented along the tentacles. All of the rods bear low sparse granulation. The pinnular scales (Figure 14e) are 0.08–0.12 mm in length, about 0.005 mm in thickness, and have a L:W of 3.5–4.5. The coenenchymal scales (Figures 14a, f) are 0.13–0.17 mm in length, about 0.01 mm in thickness, and have a H:W of 3.5–5.0. They are longitudinally oriented along the branch axis.

######## Comparisons.

Having rods in its body wall and tentacles places *C. midas* in *Chrysogorgia* Group A, the largest of the four groups of *Chrysogorgia*, consisting of 38 species (Table 1). *C. midas* is the only species in this group to have a 1/3L branching formula, this formula being much more common in Group C and in one species of Group B (see Cairns 2001).

######## Etymology.

Named “*midas*” (from the Greek Midas, the mythical king at whose touch everything turned to gold) in allusion to the golden luster of the branch axis, characteristic of the genus.

####### Chrysogorgia laevorsa sp. n.

Taxon classificationAnimaliaAlcyonaceaPrimnoidae

http://zoobank.org/26185B5F-FCA3-4160-A271-48FE5EC2E73D

**Figure 15.**
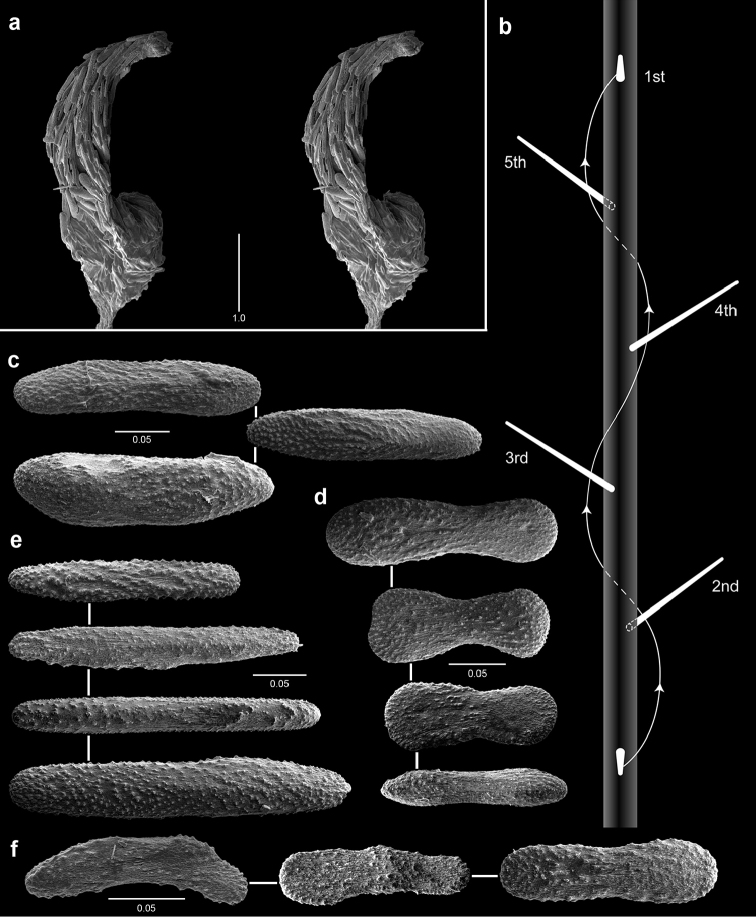
Polyps and sclerites of *Chrysogorgia laevorsa*, *JSL-I*-1929, USNM 1409029, **a** lateral stereo view of a polyp **b** diagram of 2/5R polyp arrangement, not found in *C. laevorsa* **c** upper body wall flattened rods **d** lower body wall flattened rods and waisted scales **e** tentacular flattened rods **f** pinnular scales.

Figures 3f , 15a, c–f

######## Material examined.

**Types**. Holotype: colony and SEM stubs 2320–2322, *JSL-I*-1929, USNM 1409029. Paratypes: *Alb*-2818, 13 denuded stems, USNM 1409031; *JSL-I*-1938, 1 colony, USNM 1160584.

######## Type locality.

0°14'40"N, 91°36'32"W (off Roca Redonda, Galápagos), 806 m depth.

######## Distribution.

Galápagos: Roca Redonda, west of Santa Cruz, 717–806 m deep; Cocos Islands, 614–785 m deep.

######## Description.

The colony is bottlebrush in shape (Figure 3f), the holotype 11 cm tall and 6 cm in maximum diameter, having a basal branch diameter of 2.3 mm. Branching is sympodial, the branching formula being consistently 2/5L. The orthostiche interval varies from 10 to 23 mm, the shorter intervals near the base of the colony, the higher intervals near the top. The length of the internodes on the branchlets is 3–5 mm, up to five or six nodes occurring on each branchlet; each internode supports two polyps. The polyps are 1.5–1.9 mm in length and cylindrical in shape, with a slightly swollen base (Figure 15a). The axis is metallic gold in color tinged with a greenish hue.

The upper body wall sclerites (Figure 15c), those associated with the cylindrical part of the polyp, consist of slightly flattened rotund rods that are 0.19–0.24 mm in length and having a L:W of 3.5–4.7. They are straight, longitudinally oriented, and uniformly covered with very small granules. Distal to the body wall are the tentacles, which may compose as much as half of the polyp length. They are also composed of slightly flattened rods (Figure 15c), but these rods are longer and more slender, 0.25–0.32 mm in length, with a L:W of 5.1–9.0. Like the upper body wall rods, they are straight and similarly granulated. The pinnular scales (Figure 15f) are smaller (0.073–0.16 mm in length) curved platelets, having a L:W of 3.4–4.5. The swollen base of the polyp is covered with flattened rods similar in shape to those of the tentacles (Figure 15d), as well as waisted scales 0.17–0.21 mm in length and having a L:W of 2.4–3.4. Otherwise, the coenenchyme seems to be devoid of sclerites.

######## Comparisons.

Having rods in its body wall and tentacles places *C. laevorsa* in *Chrysogorgia* Group A, the largest of the four groups of *Chrysogorgia*, having 38 species. *Chrysogorgia laevorsa* is the only species in this group to have a 2/5L branching formula, whereas 14 species in this group have a 2/5 R formula (Figure 15b). The 2/5L formula is found in only four species of *Chrysogorgia*, all belonging to Group C.

######## Etymology.

Named *laevorsa* (from the Latin *laevorsus*, meaning “towards the left”) in allusion to the direction of the branching formula (2/5L).

###### Family Isididae Lamouroux, 1812

####### Subfamily Keratoisidinae Lamouroux, 1812

######## Genus Isidella

Taxon classificationAnimaliaAlcyonaceaIsididae

Gray, 1858

Isidella Gray 1858: 283; Kükenthal 1919: 564; 1924: 414; Bayer 1956: F222; Bayer and Stefani 1987a: 51 (key to genus); 1987b: 941 (key to genus); Bayer 1990: 207–208; Etnoyer 2008: 543; Brugler and France 2008: 126–127 (inverted gene order); Watling et al. 2011: 76, fig. 2.11 (map); Dueñas et al. 2014: 20.

######### Type species.

*Isis elongata* Esper, 1788, by monotypy.

######### Diagnosis.

Colonies sparsely branched, dichotomously or trichotomously from nodes, resulting in a uniplanar colony; internodes long and hollow. Polyps non-retractile, cylindrical, armed with stout needles placed longitudinally in body wall. Spiny pharyngeal rodlets present.

######### Distribution.

Northeast Atlantic (including Mediterranean), New England Seamounts, eastern Pacific from California to Alaska, Hawaii, Galápagos, 400–2593 m deep (France 2007).

######### Remarks.

Including the species described below, there are currently six species in the genus, three of which occur in the Pacific. The genus was most recently keyed and discussed by Bayer (1990).

Using one or two mitochondrial genes and six species (most of them undescribed), France (2007) and Dueñas et al. (2014: Figure 2) indicated that *Isidella* was not monophyletic. Both papers imply that branching pattern, which has traditionally been used to distinguish keratoisidinine genera, is not a reliable character.

######## Isidella tenuis sp. n.

Taxon classificationAnimaliaAlcyonaceaIsididae

http://zoobank.org/3B9D476D-BB64-4348-B0F9-7883933E0375

Figures 3g , 16

Isidella sp. Breedy and Cortés 2008: 73, 76.

######### Material examined.

**Types**. Holotype: colony and SEM stubs 2355–2358, *JSL-I*-1942, USNM 89382. Paratypes: *JSL-I*-1942 4 colonies, USNM 1423001.

######### Type locality.

5°34.6'N, 87°04.25'W (off Cocos Island), 606–628 m deep.

######### Distribution.

Known only form the type locality.

######### Description.

**Figure 16.**
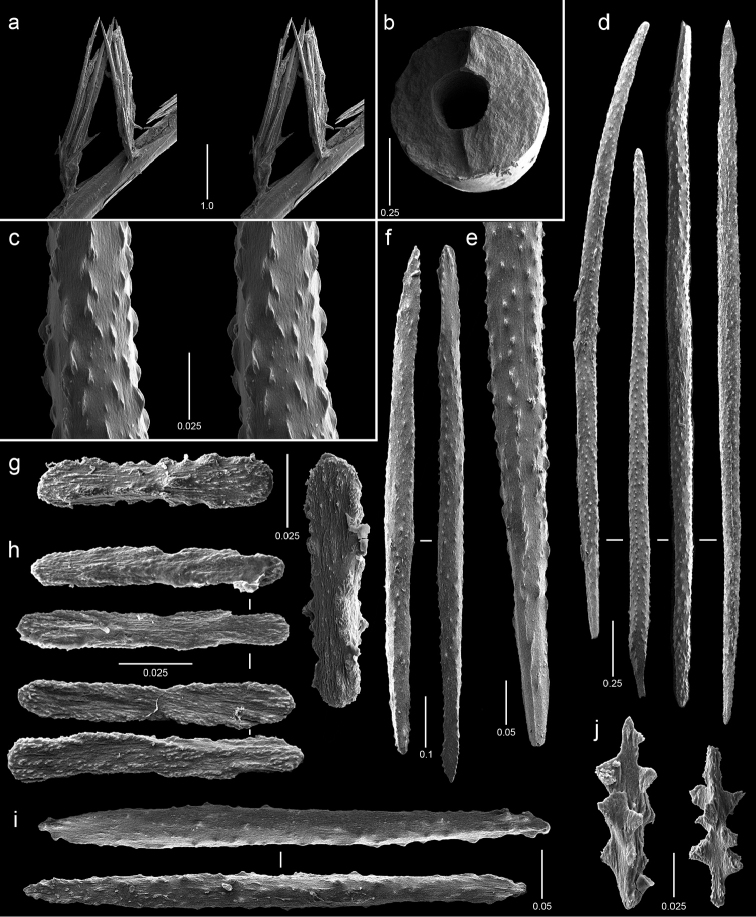
Polyps and sclerites of *Isidella tenuis*, holotype, *JSL-I*-1929, USNM 89382, **a** lateral stereo view of two polyps **b** cross section of internode showing hollow core **c** stereo view of ridged ornamentation of body wall needles **d** upper body wall needles **e** enlargement of upper body wall needle **f** lower body wall needles **g** two coenenchymal scales **h** tentacular platelets **i** basal body wall needles **j** pharyngeal rodlets.

The colony is uniplanar, the largest specimen (the holotype, Figure 3g) measuring 18 cm in height and 7 cm in width, with a basal branch diameter of 1.7 mm. The holotype shares a thin basal encrustation with another specimen (a paratype). Branching is always dichotomous from nodes, the internodes ranging from 9–14 mm in length. The internodes are white, not longitudinally grooved, and hollow, the central canal (Figure 16b) constituting about 35–40% of the branch diameter.

The polyps are uniserially placed (Figure 16a), their bases about 4–6 mm apart, but because of the length of the upturned polyps there is only 1–3 mm between adjacent polyps. The polyps are cylindrical and slender (Figure 16a), up to 3.3 mm in length and about 0.5 mm in diameter. Most of each polyp consists of eight elongate (up to 2.9 mm, L:W = 26–31), straight, cylindrical needles (Figure 16d), their pointed tips projecting beyond the tentacles. The sclerites bear numerous short (22–26 µm in length) ridges about 5 µm in height, which are arranged longitudinally on the sclerite (Figure 16e). Toward the base of the polyp are several shorter needles 0.95–1.0 mm in length (L:W = 17–19), these needles (Figure 16f) also being cylindrical but having blunt, flattened tips. Directly adjacent to the coenenchyme are also several even shorter needles (0.5 mm in length, L:W = about 13), also with flattened, blunt tips (Figure 16i). These two smaller size classes of needles allow for some flexibility of the polyp where it attached to the branch. The tentacular platelets (Figure 16h) are numerous, consisting of flat, blunt-tipped sclerites 0.095–0.1 mm in length and having a L:W of about 7. Their flat surfaces are fairly smooth, covered by small granules and low ridges. The pharyngeal sclerites (Figure 16j) are small (0.072–0.10 mm in length) rodlets that bear relatively tall spines. The coenenchymal sclerites (Figure 16g) are quite rare, consisting of flat, blunt-tipped scales (like those of the tentacles), but larger: 0.15–0.18 mm in length (L:W = 4–5).

######### Comparisons.

*Isidella tenuis* differs from *I. trichotoma* Bayer, 1990 (Hawaii, 1920 m) in having dichotomous branching, much smaller coenenchymal and body wall sclerites, and differently shaped pharyngeal sclerites. *Isidella tenuis* differs from *I. tentaculatum* Etnoyer, 2008 (California to Alaska, 720–1050 m) in having needle-shaped body wall scales, differently shaped pharyngeal scales, smaller polyps, uniserial polyps, and blunt-tipped body wall sclerites.

######### Etymology.

Named “*tenuis*” (Latin for thin) in reference to the slender polyps of the species.

## Supplementary Material

XML Treatment for Callozostron

XML Treatment for Callozostron carlottae

XML Treatment for Callogorgia

XML Treatment for Callogorgia galapagensis

XML Treatment for Callogorgia kinoshitai

XML Treatment for Calyptrophora

XML Treatment for Calyptrophora agassizii

XML Treatment for Calyptrophora reedi

XML Treatment for Narella

XML Treatment for Narella ambigua

XML Treatment for Paracalyptrophora

XML Treatment for Paracalyptrophora enigma

XML Treatment for Plumarella

XML Treatment for Plumarella (Faxiella) abietina

XML Treatment for Parastenella

XML Treatment for Parastenella pomponiae

XML Treatment for Chrysogorgia

XML Treatment for Chrysogorgia scintillans

XML Treatment for Chrysogorgia midas

XML Treatment for Chrysogorgia laevorsa

XML Treatment for Isidella

XML Treatment for Isidella tenuis

## Appendix 1

**Table:**

Station	Latitude / Longitude	Depth (m)	Date
JSL-I				
1912	0°21.877'S, 90°15.747'W	806	Nov 14 1986
1915	1°17.2'S, 89°48.7'W	650	Nov 15 1986
1916	1°18.715'S, 89°48.809'W	545–562	Nov 16 1986
1922	0°23.683'N, 90°26.341'W	475–578	Nov 19 1986
1927	0°10.50'S, 90°53.25'W	708–782	Nov 21 1986
1929	0°14.675'N, 91°36.526'W	804	Nov 23 1986
1930	0°03.057'S, 91°33.927'W	333–478	Nov 23 1986
1931	0°10.303'S, 91°24.675'W	441–528	Nov 24 1986
1933	0°17.072'S, 91°40.208'W	663–788	Nov 25 1986
1934	0°15.16'S, 91°27.93'W	252–308	Nov 25 1986
1935	0°29.737'S, 91°23.806'W	391–488	Nov 26 1986
1938	5°24.49'N, 87°09.83'W	614–785	Nov 30 1986
1942	5°34.60'N, 87°04.25'W	606–628	Dec 2 1986
3902	1°18.763'S, 89°48.82'W	554	Oct 17 1995
3907	0°20.517'N, 90°23.788'W	530–576	Oct 19 1995
3912	0°08.581'N, 91°23.708'W	489	Oct 21 1995
3925	0°17.100'S, 91°05.046'W	768	Oct 28 1995
3930	0°29.749'S, 90°13.981'W	450	Oct 30 1995
JSL-II				
3108	0°24.00'N, 90°26.5'W	1545	Jul 21 1998
USFWS Albatross				
2818	0°29'S, 89°54'30"W	717	Apr 15 1888
3399	1°07'00"N, 81°04'W	3182	Mar 24 1891
3403	0°58'30"S, 89°17'W	700	Mar 28 1891
3404	1°03'S, 89°28'W	704	Mar 28 1891
3406	0°16'S, 90°21'30"W	1008	Apr 3 1891
3410	0°19'N, 90°34'W	805	Apr 3 1891
4153	23°05'N, 161°52'W	1760–1837	Aug 5 1902
4357	32°N, 117°W	245–284	Mar 15 1904
4530	36°45'N, 121°55'W	1381–1752	May 27 1904
4537	36°45'N, 121°55'W	1575–1942	May 31 1904
R/V Gilliss				
21	7°11'N, 79°16'W	1463	Jan 18 1972
E/V Nautilus				
NA64–77–01	0°23.40'S, 91°52.98'W	3381	Jul 3 2015
NA64–125–01	0°22.60'S, 90°40.05'W	446	Jul 6 2015
NA64–126–01	0°22.59'S, 90°49.06'W	445	Jul 6 2015
