# Corrigenda: Cairns SD (2018) Deep-Water Octocorals (Cnidaria, Anthozoa) from the Galápagos and Cocos Islands. Part 1: Suborder Calcaxonia. ZooKeys 729: 1–46. https://doi.org/10.3897/zookeys.729.21779

**DOI:** 10.3897/zookeys.735.23890

**Published:** 2018-02-06

**Authors:** Stephen D. Cairns

**Affiliations:** 1 Department of Invertebrate Zoology, National Museum of Natural History, Smithsonian Institution, P. O. Box 37012, MRC 163, Washington, D.C. 20013-7012, USA

Regarding the specimens collected by the *E/V Nautilus*, I would like to further thank the Galapagos National Park Directorate for providing joint collection permit PC-45-15 in collaboration with the Charles Darwin Foundation, and thank the government of Ecuador, the Ecuadorian Navy, and its Oceanographic Institute (INOCAR) for their support and permission to operate in their territorial waters. This publication is contribution number 2189 of the Charles Darwin Foundation for the Galapagos Islands.

